# An Explainable XGBoost-Based Framework for IoT Attack Detection with Unseen Attack Family Evaluation

**DOI:** 10.3390/s26103005

**Published:** 2026-05-10

**Authors:** Ruei-Jan Hung

**Affiliations:** Department of Electronic Engineering, Cheng Shiu University, Kaohsiung 833301, Taiwan; k6736@gcloud.csu.edu.tw

**Keywords:** Internet of Things, IoT attack detection, intrusion detection, explainable artificial intelligence, XAI, unseen attack family evaluation, analyst-centered explanation

## Abstract

The rapid growth of the Internet of Things (IoT) has introduced significant cybersecurity challenges due to the heterogeneity, scale, and limited protection capability of connected devices. Although machine learning has been widely adopted for IoT intrusion detection, many existing studies still rely primarily on closed-world evaluation settings, unequal baseline comparison budgets, fixed decision thresholds, and limited integration of explainability into model assessment. To address these issues, this paper proposes an explainable XGBoost-based framework for IoT attack detection with unseen attack family evaluation using the large-scale CICIoT2023 dataset. In the proposed framework, IoT traffic is formulated as a binary classification task that distinguishes benign from malicious flows. The study integrates two complementary evaluation protocols: (1) closed-world stratified 10-fold cross-validation for in-distribution performance assessment and (2) unseen attack family evaluation, in which one malicious family is excluded from training and used only for testing under a zero-day-like but single-dataset condition. A fair-budget experimental design is adopted to compare seven representative models under the same training budget, including default XGBoost, optimized XGBoost, Random Forest, LightGBM, CatBoost, Logistic Regression, and a simple multilayer perceptron. To improve reproducibility and operational validity, the revised framework further reports the sampling strategy, split-overlap audit, XGBoost hyperparameter search protocol, repeated unseen-family evaluation, validation-based threshold calibration under fixed-FAR constraints, cost-sensitive threshold analysis, and XGBoost-native SHapley Additive exPlanations (SHAP) compatible feature contribution analysis. The closed-world results show that tree-based ensemble methods clearly outperform the linear and shallow neural baselines. Random Forest achieves the highest closed-world macro-F1 of 0.9713, followed by LightGBM with 0.9602 and optimized XGBoost with 0.9566. In the fair-budget unseen-family setting under the default threshold, Random Forest again obtains the highest mean macro-F1 of 0.8433 and the lowest false negative rate (FNR) of 0.0712, but it also produces a substantially higher false alarm rate (FAR = 0.0536). By contrast, optimized XGBoost provides a lower-FAR default operating point, achieving a mean macro-F1 of 0.8194, Matthews correlation coefficient (MCC) of 0.7067, FAR of 0.0086, and FNR of 0.2996. Repeated unseen-family experiments over five random seeds confirm the same trade-off: Random Forest provides stronger recall-oriented detection, whereas optimized XGBoost provides a lower-FAR default operating point. After validation-based threshold calibration at an approximate FAR target of 0.01, Random Forest achieves the strongest calibrated recall-oriented performance, with macro-F1 of 0.8754, MCC of 0.7757, FNR of 0.2000, and attack recall of 0.8000. Optimized XGBoost remains competitive at the same FAR target, with macro-F1 of 0.8323, MCC of 0.7193, FNR of 0.2760, and attack recall of 0.7240. The explainability analysis indicates that the optimized XGBoost detector relies mainly on TCP control-flag, temporal, and packet-statistical features, with rst_count, IAT, urg_count, Tot size, Number, Header_Length, and Magnitude among the most influential variables. Local contribution tables for representative true-positive, false-positive, false-negative, and true-negative cases further improve the readability of the explanation results and confirm that native pred_contribs reconstructs the model margin with negligible numerical error. Overall, the results show that the most appropriate model depends on the deployment objective: Random Forest is preferable when minimizing missed attacks under a calibrated FAR constraint is prioritized, whereas optimized XGBoost remains a strong primary model for an explainable low-FAR XGBoost-based framework that emphasizes scalability, operational conservativeness, and native contribution-based interpretation.

## 1. Introduction

### 1.1. Research Background and Motivation

The rapid expansion of the Internet of Things (IoT) has transformed modern digital infrastructures by connecting large numbers of heterogeneous devices across smart homes, healthcare systems, industrial control networks, intelligent transportation, and urban sensing environments. As IoT adoption continues to grow, the resulting ecosystems increasingly combine massive connectivity, continuous data generation, and constrained endpoint protection capabilities. While this connectivity enables automation, monitoring, and service optimization, it also enlarges the attack surface and exposes IoT networks to a broad range of cyber threats. The recent literature consistently emphasizes that IoT environments remain especially vulnerable because devices are often heterogeneous, resource-constrained, weakly managed, and deployed for long operational lifecycles, making conventional security mechanisms difficult to apply effectively [1,2,3].

To address these threats, intrusion detection systems (IDSs) and network intrusion detection systems (NIDSs) have become a major line of defense in IoT cybersecurity. In recent years, machine learning and deep learning techniques have been widely investigated for IoT attack detection because they can learn complex decision boundaries from large-scale traffic data and support automated malicious traffic screening. However, recent reviews and large-scale empirical studies also indicate that existing IoT IDS research still faces several unresolved challenges, including model robustness under realistic conditions, data imbalance, computational constraints, explainability, and deployment suitability in resource-limited environments [2,3,4].

The emergence of large and realistic IoT cybersecurity datasets has significantly improved the empirical basis for this research area. Among them, CICIoT2023 is particularly important because it was designed as a real-time large-scale benchmark for IoT security research and includes benign traffic together with 33 attacks grouped into seven major categories, generated in an environment involving 105 IoT devices [1]. Such characteristics make CICIoT2023 especially suitable for studying large-scale IoT attack detection under heterogeneous traffic conditions.

Within this broader context, tree-based ensemble learning remains one of the most effective approaches for structured intrusion detection data. XGBoost has been especially influential because of its scalable gradient boosting formulation, regularization mechanisms, and consistently strong performance on large tabular learning problems [5]. More recent IoT-focused work also shows that XGBoost-based intrusion detection frameworks can remain competitive when interpretability and security analysis are considered together [6]. At the same time, explainable artificial intelligence (XAI) has become increasingly important in intrusion detection because security analysts often need to understand why traffic is classified as malicious, which features dominate the decision process, and why false positives or false negatives occur [7,8,9].

Accordingly, this study is motivated by the need for an IoT attack detection framework that is not only accurate under standard benchmark evaluation, but also methodologically reproducible, operationally meaningful, threshold-aware, and interpretable. In particular, it is necessary to examine whether a high-performing XGBoost-based detector remains useful under stricter unseen-family generalization settings, whether its behavior changes under false-alarm-constrained threshold calibration, and whether its decisions can be explained in a form that supports cybersecurity analysis.

### 1.2. Research Gap and Problem Statement

Although many IoT intrusion detection studies report excellent classification results, a substantial portion of the literature still evaluates models primarily under conventional closed-world settings, such as random train–test splits or standard cross-validation. These protocols are useful for measuring in-distribution discrimination capability, but they do not necessarily reflect how a detector behaves when it encounters attack behaviors that were not explicitly represented during training. In particular, a detector may achieve near-saturated accuracy or F1-score when training and testing samples share the same family-level distribution, while still performing less reliably when an entire malicious family is absent from training.

A second limitation is that many baseline comparisons are not performed under equal training budgets or clearly documented sampling strategies. In large-scale IoT intrusion detection, models may appear superior simply because they receive more training samples, more favorable preprocessing, or more extensive tuning. Without specifying whether the training subset was drawn randomly or stratified, whether sampling was repeated over multiple seeds, and whether train–test overlap was audited, the reproducibility and fairness of the comparison remain limited. This issue becomes especially important for CICIoT2023 because the dataset is extremely imbalanced, with attack traffic accounting for approximately 97.65% of all samples.

A third limitation concerns operational decision thresholds. Many studies report results at a default threshold, even when they make practical claims about false alarm control. However, different models may behave very differently after threshold calibration. A model with a high default false alarm rate may become competitive after validation-based threshold adjustment, while a low-FAR model at the default threshold may exhibit a higher false negative rate when compared at the same FAR target. Therefore, a deployment-oriented evaluation should not rely only on macro-F1 or accuracy at a fixed threshold; it should also examine FAR/FNR trade-offs, recall under fixed-FAR constraints, and cost-sensitive operating points.

A fourth gap concerns explainability. Although explainable IDS research has grown rapidly, many studies still treat XAI primarily as a post hoc visualization step rather than as an analytical component integrated with rigorous evaluation. Feature attribution can help inspect model behavior, but operational explainability also requires clearer local summaries, diagnostic interpretation of false positives and false negatives, and the acknowledgement of the difference between model-centered attribution and user-centered explanation. In cybersecurity operations, analysts often need evidence that helps them understand why an alert was raised, why an attack was missed, and whether the explanation is meaningful in relation to traffic behavior.

These limitations lead to the central problem addressed in this study: how to design an IoT attack detection framework that combines strong tabular-learning performance, fair and reproducible model comparison, unseen-family robustness assessment, threshold-aware operational evaluation, and interpretable decision analysis. To address this problem, this paper formulates IoT intrusion detection as a binary malicious-versus-benign classification task on CICIoT2023 and evaluates the resulting models under closed-world, unseen-family, repeated-sampling, threshold-calibrated, and explainability-oriented settings.

### 1.3. Research Objectives and Contributions

The main objective of this study is to develop and evaluate an explainable XGBoost-based framework for IoT attack detection under both standard benchmark conditions and more deployment-oriented unseen-family conditions. Rather than assuming that a single model is universally optimal, this study examines how model preference changes when the deployment objective shifts from default-threshold aggregate performance to low-FAR operation, recall under fixed-FAR constraints, or cost-sensitive threshold selection.

To achieve this objective, this paper makes the following contributions.

First, this study proposes an explainable XGBoost-based framework for large-scale IoT attack detection using the CICIoT2023 benchmark dataset. The framework focuses on binary malicious traffic detection and is designed for structured network-flow features derived from a realistic IoT environment.

Second, this study establishes a fair-budget comparative setting in which seven representative models are trained under the same 2,000,000 sample budget. The sampling process is explicitly described as stratified sampling without replacement from the training candidate pool, and the same sampled subset is used for all models within each run. This design reduces methodological bias and makes it easier to attribute observed differences to model behavior rather than unequal data access.

Third, this study introduces an unseen attack family evaluation protocol in which each malicious family is excluded from training and used only for testing. Benign traffic used for testing is also separated from training and validation. The protocol is repeated across nine malicious families and five random seeds, producing 45 unseen-family runs for the supplementary repeated evaluation. Split-overlap audits confirm that no sample overlap exists between fitting, validation, and test partitions.

Fourth, this study adds threshold-aware operational evaluation. In addition to default-threshold results, validation-based threshold calibration is performed under fixed-FAR targets, and cost-sensitive threshold selection is used to examine different deployment risk preferences. This addition directly addresses the practical distinction between minimizing false alarms and minimizing missed attacks.

Fifth, this study reports a reproducibility-oriented hyperparameter search protocol for optimized XGBoost, including search space, validation strategy, selection criterion, and random seed handling. This strengthens the reproducibility of the optimized model and clarifies that model tuning is separated from final unseen-family testing.

Sixth, this study integrates XGBoost-native SHAP-compatible feature contribution analysis into the experimental framework. The revised explainability analysis includes both global feature contribution ranking and local contribution tables for representative true-positive, false-positive, false-negative, and true-negative cases. The local reconstruction check confirms that native pred_contribs satisfies additive reconstruction in margin space, while the manuscript explicitly clarifies that these contributions should not be interpreted as probability-space SHAP values.

Overall, the contribution of this study lies not merely in presenting another accurate IoT classifier, but in combining fair-budget comparison, repeated unseen-family assessment, threshold-calibrated operational evaluation, and explainable model analysis into a unified framework for deployment-aware IoT cybersecurity research.

### 1.4. Organization of This Paper

The remainder of this paper is organized as follows. Section 2 reviews related work on IoT intrusion detection, benchmark datasets, tree-based learning, explainable AI, unseen-family evaluation, and deployment-oriented IDS assessment. Section 3 presents the proposed methodology, including dataset preparation, binary problem formulation, attack family grouping, fair-budget sampling, model construction, hyperparameter search, evaluation protocols, threshold calibration, performance metrics, and explainability design. Section 4 reports the experimental results under closed-world, unseen-family, repeated-sampling, fixed-FAR, cost-sensitive, full-scale, and XAI-based analyses. Section 5 discusses the implications of the findings, especially the deployment-dependent trade-off between FAR and FNR, the meaning of threshold calibration, the role of explainability, and the limitations of the present study. Section 6 concludes the paper and outlines future research directions.

## 2. Related Work

### 2.1. IoT Intrusion Detection and Benchmark Datasets

Intrusion detection in IoT environments has become a major research topic because IoT devices typically operate under severe constraints in computation, memory, energy, and patch management, while simultaneously being deployed in highly heterogeneous and continuously connected environments. As a result, IoT ecosystems are exposed to a broad range of threats, including volumetric denial-of-service attacks, reconnaissance, spoofing, credential abuse, and malware-driven botnet behavior. Recent surveys consistently argue that IoT intrusion detection remains challenging not only because of the diversity of devices and protocols, but also because of deployment realism, adaptation requirements, and the tension between detection performance and lightweight operational constraints [2,3]. Accordingly, intrusion detection has evolved into one of the most actively studied defensive mechanisms in the IoT security domain.

A large body of recent work has applied machine learning and deep learning to IoT traffic classification and attack detection. Some studies have focused on optimizing feature engineering pipelines for IoT network intrusion detection, especially under the need to reduce computation and inference time while preserving detection quality [2,4]. Others have investigated deep models, residual architectures, transfer learning, federated learning, and stream-oriented security analytics in order to improve adaptability across heterogeneous IoT environments [10,11,12,13]. These developments indicate that IoT intrusion detection is no longer limited to simple classification accuracy; instead, the research focus has broadened toward scalability, real-time feasibility, generalization, distributed learning, and interpretability.

The quality and realism of the underlying benchmark datasets play a decisive role in evaluating such systems. Among recent public benchmarks, CICIoT2023 has emerged as one of the most important datasets for large-scale IoT cybersecurity research. CICIoT2023 was constructed in a real-time IoT testbed, involving 105 devices and includes benign traffic, together with 33 attacks categorized into seven major groups [1]. Compared with smaller or less diverse benchmarks, CICIoT2023 provides a more realistic basis for evaluating modern attack detection models under large-scale heterogeneous IoT conditions. Because of these characteristics, the dataset is well suited for both binary malicious-versus-benign detection and more demanding studies of attack family behavior.

Nevertheless, benchmark usage alone does not guarantee realistic evaluation. Many IoT IDS studies still rely primarily on closed-world settings, where training and testing data are sampled from the same global data distribution. Such settings are useful for measuring in-distribution discrimination capability, but they may overestimate how well a detector handles unfamiliar malicious behaviors in practice. Recent reviews explicitly note that the field still needs stronger attention to adaptive, realistic, and operationally grounded evaluation protocols [3]. This limitation motivates evaluation strategies that move beyond ordinary random splitting and better approximate the uncertainty faced by deployed IoT security systems.

### 2.2. Tree-Based Learning for IoT Attack Detection

Among machine learning methods for structured intrusion detection data, tree-based ensemble models remain especially competitive. The classical XGBoost framework introduced scalable gradient tree boosting with regularization, sparsity-aware optimization, and efficient learning for large tabular datasets and has since become one of the most influential methods in applied machine learning [5]. In intrusion detection contexts, this model family is attractive because network-flow features are typically numerical, tabular, heterogeneous in distribution, and rich in nonlinear interactions, which are precisely the conditions under which boosting and tree ensembles often perform well.

Recent IoT intrusion detection studies continue to confirm the practical strength of tree-based approaches. XGBoost has been used as a high-performance baseline as well as a primary model in explainable intrusion detection pipelines [6]. Random Forest remains a strong classical comparator due to its stability, resilience to noise, and robust empirical performance on structured attack detection tasks. LightGBM and CatBoost further extend the family of boosting-based baselines by emphasizing efficiency, gradient-based optimization, and stable training behavior. Together, these methods form a natural comparison group for IoT attack detection because they can model complex nonlinear decision boundaries while remaining compatible with large-scale tabular feature sets.

At the same time, model performance cannot be interpreted independently of feature engineering, sampling strategy, computational constraints, and decision thresholds. Comparative IoT IDS studies emphasize that feature selection and feature extraction can alter both detection quality and computational cost [2,4]. Similarly, constrained-device studies highlight that strong benchmark results may not translate directly into realistic deployment suitability when training or inference cost is high [10]. Therefore, a rigorous comparison among tree-based models should control for training budget, random seed handling, threshold selection, and evaluation protocol, rather than implicitly favoring one method through more generous data usage or tuning effort.

Although deep learning has become increasingly popular for intrusion detection, this study does not aim to exhaustively benchmark complex neural architectures such as CNNs, RNNs, LSTMs, or transformers. The main experimental focus is large-scale tabular network-flow classification under a fair and reproducible budget, where tree-based methods remain highly competitive, computationally efficient, and directly compatible with feature contribution analysis. A simple multilayer perceptron is included as a lightweight neural baseline to represent a shallow neural alternative under the same budget. More complex sequential or representation-learning architectures are left for future work because they often require different input representations, temporal windowing assumptions, hyperparameter search spaces, and computational budgets, which would weaken the controlled nature of the present fair-budget comparison.

### 2.3. Explainable Artificial Intelligence for Intrusion Detection

As intrusion detection systems become more accurate and complex, explainability has become increasingly important in cybersecurity. In operational environments, analysts often need more than a simple malicious-versus-benign decision. They must understand why an alert was raised, which traffic features drove the decision, whether a false positive reflects recurring benign behavior that resembles attack traffic, and whether a false negative exposes a systematic blind spot. Recent surveys on explainable AI for cybersecurity and traffic analysis stress that interpretability is important not only for model transparency but also for analyst trust, error diagnosis, compliance, and operational validation [8,14].

Among the most influential interpretability methods, SHAP provides an additive feature attribution framework grounded in Shapley-value theory [7]. SHAP-style explanations have become widely used because they support both global and local interpretation. At the global level, they identify features that strongly influence the model overall. At the local level, they explain why a specific instance was classified as malicious or benign. These properties are especially valuable in intrusion detection, where analysts may wish to inspect both general traffic patterns and particular alert cases.

Recent explainable IDS studies have extended this line of work in several directions. Some works have proposed general explainable IDS frameworks for improving transparency in network intrusion detection [15,16]. Others have focused specifically on IoT settings, combining explainability with deep learning, rule induction, anomaly detection, or stream-based intrusion detection [6,9,17,18,19,20,21,22,23]. These studies collectively show that explainability is no longer a peripheral concern; rather, it has become a central requirement for making intrusion detection models auditable and practically usable.

However, an important limitation remains. In many cases, explainability is still treated mainly as an auxiliary visualization step after model training. That is, the main research contribution remains a performance-oriented classifier, while XAI is appended mainly to produce feature importance plots. Fewer studies integrate explainability with rigorous evaluation of model behavior across true positives, false positives, false negatives, and true negatives, or use it to support a more systematic understanding of generalization under challenging threat conditions. This is one of the key gaps addressed in the present work. Instead of treating XAI as a cosmetic addition, this study incorporates XGBoost-native SHAP-compatible feature contribution analysis into the experimental pipeline to examine both global feature behavior and representative success and error cases.

It is also important to distinguish model-centered attribution from user-centered explainability. Feature attribution explains which variables contributed to a model output, but it does not by itself guarantee that the explanation is actionable for a security analyst. In practical IoT security operations, a useful explanation should help the analyst understand whether an alert is plausible, what traffic behavior caused the decision, and whether additional investigation is warranted. Therefore, the present study interprets contribution values as diagnostic model evidence rather than complete human-centered explanations. The revised analysis adds local contribution summary tables to complement waterfall plots and discusses how such summaries can support analyst review while acknowledging that fully user-centered explanation design remains a future research direction.

### 2.4. Unseen Attacks, Generalization, and Evaluation Realism

A growing body of recent work argues that conventional IDS benchmarks do not always capture the operational difficulty of detecting previously unseen or weakly represented attacks. Standard cross-validation is useful for estimating general discrimination performance, but it does not directly evaluate the capacity of a model to identify malicious traffic from attack types or families absent during training. This problem becomes especially important in IoT environments, where new devices, changing traffic profiles, and evolving attack strategies can quickly invalidate assumptions learned from historical data.

Recent studies on zero-day-like, few-shot, adaptive, and meta-learning-oriented intrusion detection have emphasized that realistic evaluation must account for distribution shift, operational false alarm constraints, and the model’s ability to generalize under incomplete prior knowledge [23,24,25]. In particular, operationally constrained zero-day intrusion detection research highlights that the detector’s usefulness depends not only on raw detection power, but also on whether performance can be maintained under acceptable false positive rates. Similarly, adaptive and meta-learning-oriented IDS research reflects the broader recognition that intrusion detection should be evaluated in a way that better approximates unknown or evolving attacks rather than assuming that all relevant malicious categories are already represented during training [25].

Despite this trend, relatively few IoT IDS studies combine large-scale benchmark evaluation with an explicit unseen-family design. Most work still reports results from binary or multiclass random split experiments, which may blur the distinction between in-distribution performance and genuine robustness to unfamiliar malicious behavior. Consequently, there remains a gap between high benchmark performance and operational confidence.

The present study addresses this issue by introducing an unseen attack family evaluation protocol on top of conventional closed-world cross-validation. In this design, one malicious family is fully excluded from training and used only for testing. This does not constitute a fully open-world or cross-dataset zero-day benchmark, but it provides a more stringent estimate of how well the detector generalizes when previously unseen attack families are encountered. By combining this protocol with fair-budget comparison, threshold calibration, and XAI analysis, the proposed framework seeks to align methodological rigor more closely with operational relevance.

### 2.5. Research Positioning

Based on the above review, four limitations can be identified in the current literature. First, although IoT intrusion detection has matured considerably, many studies still rely mainly on closed-world evaluation settings and therefore provide limited insight into how detectors behave when facing previously unseen malicious families. Second, comparative studies often do not strictly control for training budget, sampling strategy, and random seed handling, which weakens the interpretability of model ranking. Third, many studies report performance only at a default decision threshold even when they make deployment-oriented claims about false alarm control. Fourth, explainability is frequently included only as a post hoc feature visualization rather than as an integrated analytical tool for understanding model behavior, error patterns, and operational trade-offs.

This study is positioned at the intersection of these issues. Unlike research that focuses only on conventional random-split performance, this paper combines closed-world cross-validation with unseen-family evaluation. Unlike studies that compare models under different effective training conditions, this work introduces a fair-budget comparison across seven representative baselines and explicitly documents the sampling strategy. Unlike default-threshold-only evaluations, this work adds validation-based fixed-FAR calibration and cost-sensitive threshold analysis. Finally, unlike performance-oriented studies that append explainability only as a supplementary illustration, this paper uses XGBoost-native SHAP-compatible feature contribution analysis to examine global feature importance and local TP, FP, FN, and TN cases.

Accordingly, the research contribution of this work does not lie solely in achieving high IoT attack detection accuracy. Instead, it lies in proposing a more rigorous and practically informative framework that combines large-scale tabular learning, controlled baseline comparison, repeated unseen-family robustness assessment, threshold-aware operational evaluation, and explainable security analysis in a unified design. Table 1 summarizes the positioning of representative related studies and the present work.

## 3. Methodology

### 3.1. Overall Research Framework

This study proposes an explainable XGBoost-based framework for IoT attack detection under both conventional closed-world evaluation and a more challenging unseen attack family setting. The overall workflow consists of eight major stages: dataset loading, preprocessing and binary labeling, attack family grouping, fair-budget sampling, model training, protocol-specific evaluation, threshold calibration, and explainability analysis. The complete framework was implemented on the CICIoT2023 dataset and designed to support fair-budget multi-model comparison, supplementary full-scale optimized XGBoost evaluation, repeated unseen-family robustness assessment, and XAI-based interpretation.

First, the CICIoT2023 dataset was loaded from 169 partitioned files, resulting in a final dataset containing 46,686,579 traffic instances and 46 predictive features. Each record was mapped into a binary label indicating benign or attack traffic. The original attack labels were also grouped into higher-level attack families to support unseen-family evaluation. Based on the processed dataset, the study conducted two main experimental tracks. The first track used fair-budget multi-model comparison, where all candidate models were trained under the same 2,000,000-sample training budget. The second track used a supplementary full-scale optimized XGBoost setting to examine whether the selected model benefited from larger-scale training beyond the shared-budget comparison.

**Figure 1:**
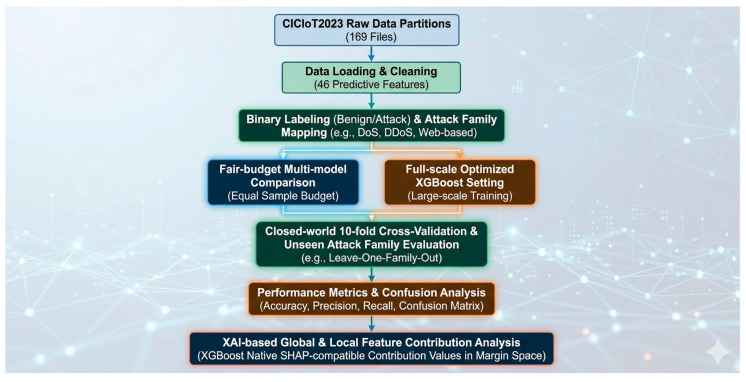
Overall workflow of the proposed explainable IoT attack detection framework.

The revised framework further includes three reviewer-driven supplementary components. First, repeated unseen-family evaluation was conducted across five random seeds to assess sampling variance. Second, validation-based threshold calibration was performed under fixed-FAR targets to examine operational performance when false alarms are explicitly constrained. Third, cost-sensitive threshold selection was used to analyze how model preference changes under different false-positive and false-negative cost assumptions. The overall workflow of the proposed framework is shown in Figure 1.

The explainability stage was conducted after model training and evaluation. Feature contribution analysis was performed using XGBoost native pred_contribs, which produces SHAP-compatible contribution values in the model margin space. This analysis was conducted at both global and local levels to identify important traffic features and to interpret representative true-positive, false-positive, false-negative, and true-negative cases.

### 3.2. Dataset Description and Preprocessing

This study employed the CICIoT2023 dataset as the experimental benchmark. The dataset was chosen because it is one of the largest publicly available benchmarks specifically designed for IoT cybersecurity research and contains both benign traffic and a wide spectrum of attack behaviors. According to the experimental artifacts used in this study, the final merged dataset consisted of 46,686,579 samples and 46 numerical flow-based features. These features included temporal characteristics, TCP flag statistics, protocol indicators, and packet-level statistical descriptors, making them suitable for large-scale tabular learning. The overall dataset scale is summarized in Table 2, and the attack family distribution is reported in Table 3.

The preprocessing stage first concatenated all 169 partition files into a single analytical dataset. The original label field was then converted into a binary target variable, where benign traffic was encoded as 0 and attack traffic was encoded as 1. The resulting class distribution was highly imbalanced, with 1,098,195 benign samples and 45,588,384 attack samples, corresponding to an attack ratio of approximately 97.65%. Because of this imbalance, the evaluation design emphasized macro-F1, balanced accuracy, MCC, FAR, and FNR rather than relying only on accuracy or PR-AUC.

To support unseen attack family evaluation, the original fine-grained attack labels were grouped into broader attack families. Based on the mapping used in this study, the ten resulting families were Benign, DDoS, DoS, Mirai, Spoofing, Recon, Web, BruteForce, BrowserHijacking, and Backdoor. Since the unseen-family protocol focuses on malicious traffic not observed during training, only the nine malicious families were used as held-out attack groups, while benign traffic was retained as the normal class in all runs.

No global feature scaling was applied before splitting the data. This decision was made to avoid preprocessing leakage. For tree-based models, feature scaling is not required and was therefore omitted. For non-tree baselines that are more sensitive to feature scale, normalization was applied only within the model-specific training pipeline after the train–test split.

The feature name “Magnitue” appears in the original CICIoT2023 feature file and was retained in the experimental code for exact column-name compatibility. In the manuscript’s discussion, this variable is referred to as “Magnitude” while preserving the original feature spelling in tables when reporting raw column names.

### 3.3. Problem Formulation

This study formulates IoT attack detection as a supervised binary classification problem. Let the traffic feature vector of the i-th sample be denoted by $x_{i}\in {\mathbb{R}}^{d}$, where d is the number of predictive features, and let the corresponding binary label be $y_{i}\in \left\{0,1\right\}$. The binary label assignment is defined as follows:

$$y_{i}=\left\{\begin{array}{ll} 0, & \;\;\;\mathrm{if}\;\mathrm{the}\;i\mathrm{-th}\;\mathrm{traffic}\;\mathrm{sample}\;\mathrm{is}\;\mathrm{benign},\\ 1, & \mathrm{if}\;\mathrm{the}\;i\mathrm{-th}\;\mathrm{traffic}\;\mathrm{sample}\;\mathrm{is}\;\mathrm{malicious}. \end{array}\right.$$

The learning objective is to train a classifier $f\left(x\right)$ that estimates the probability of malicious traffic:

$$p_{i}=P\left(y_{i}=1|x_{i}\right).$$

A predicted label is then produced by applying a decision threshold τ:

$${\hat{y}}_{i}=\left\{\begin{array}{ll} 1, & \mathrm{if}\;p_{i}\geq \tau ,\\ 0, & \mathrm{if}\;p_{i}<\tau . \end{array}\right.$$

The main closed-world and unseen-family experiments used the default threshold τ=0.5 unless otherwise specified. To address operational false alarm control, supplementary experiments also used validation-based calibrated thresholds under fixed-FAR and cost-sensitive objectives.

For the unseen attack family setting, the attack family label of each malicious sample was also used to define a more stringent generalization protocol. Let A denote the set of malicious attack families. During each unseen-family run, one family a∈A was completely excluded from training and used only for testing. This design evaluates whether the classifier can identify malicious traffic patterns from an attack family not explicitly observed during training.

### 3.4. Fair-Budget Sampling and Split Audit

The fair-budget setting used 2,000,000 training samples per model. This sample size was selected as a practical compromise between dataset scale, computational feasibility, and fair multi-model comparability. CICIoT2023 contains more than 46 million records, and training all models repeatedly on the full dataset would make repeated unseen-family, calibration, and XAI analyses computationally expensive. The 2,000,000-sample budget is large enough to preserve the dominant benign/attack structure and family diversity of the dataset while allowing all candidate models to be evaluated under identical data access conditions.

For each unseen-family run, all samples from the held-out malicious family were assigned to the test set. Benign traffic was split into training/validation and test portions, with 20% of benign samples held out for testing. Thus, the benign samples used for testing were not used for fitting or validation. Within a given random seed, the same benign test pool was reused across held-out-family runs to make FAR comparable across families. Across random seeds, the benign split was resampled to assess sampling variance.

From the remaining training candidate pool, 2,000,000 samples were drawn by stratified sampling without replacement. The sampled training budget was then split into 1,700,000 fitting samples and 300,000 validation samples. The validation subset was used for threshold calibration and hyperparameter selection where applicable. The same sampled subset and split were used for all compared models within each run, ensuring that model comparisons were not affected by unequal data exposure.

A split-overlap audit was performed for every repeated unseen-family run. Across all 45 supplementary runs, the audit confirmed zero overlap between train and test partitions, zero overlap between fitting and validation partitions, zero overlap between fitting and test partitions, and zero overlap between validation and test partitions. This audit provides explicit evidence that no training, validation, or testing leakage occurred in the repeated unseen-family experiments.

The detailed sampling and split audit results are provided in Supplementary Table S1.

### 3.5. Model Construction and Baseline Design

To assess the effectiveness of the proposed framework, this study compared optimized XGBoost against several representative machine learning baselines commonly used in tabular intrusion detection tasks. The evaluated models included default XGBoost, optimized XGBoost, Random Forest, LightGBM, CatBoost, Logistic Regression, and a simple multilayer perceptron. These models were chosen to cover a broad range of algorithmic families, including boosting-based tree ensembles, bagging-based tree ensembles, linear classification, and shallow neural networks.

The default XGBoost model served as the reference gradient boosting baseline, while the optimized XGBoost model represented the proposed primary detector for the explainable XGBoost-based framework. Random Forest was included because it is a strong classical ensemble model for tabular classification. LightGBM and CatBoost were selected as competitive gradient boosting baselines. Logistic Regression provided a linear baseline, while the simple multilayer perceptron represented a lightweight neural-network comparator.

Complex neural architectures such as CNNs, RNNs, LSTMs, and transformers were not included in the main baseline suite because this study focuses on controlled large-scale tabular flow classification under identical training budgets. Such deep architectures often require different temporal representations, sequence construction, architecture-specific tuning, and computational budgets. Including them under the same tabular setting would not provide a fair representation of their intended use. Therefore, this study treats the simple MLP as a lightweight neural baseline and leaves broader deep sequential modeling for future work. The roles of the evaluated models are summarized in Table 4, and the training-budget design is summarized in Table 5.

### 3.6. Optimized XGBoost Hyperparameter Search

To improve reproducibility, the revised study reports the optimized XGBoost configuration and the supplementary hyperparameter search protocol. The search used the 2,000,000-sample fair-budget setting, with 1,700,000 samples used for fitting and 300,000 samples used for validation. The selection criterion emphasized validation macro-F1 and MCC under the default threshold, supplemented by approximate fixed-FAR threshold calibration at FAR target = 0.01. The purpose of this protocol was to document the search space and confirm that the optimized configuration was selected without using the final unseen-family test labels.

The optimized XGBoost configuration used in the main experiments was as follows: n_estimators = 354, max_depth = 10, learning_rate = 0.170176, subsample = 0.799013, colsample_bytree = 0.808141, min_child_weight = 1, gamma = 0, reg_lambda = 1, and reg_alpha = 0. In the supplementary hyperparameter search, this configuration remained the strongest candidate among the evaluated configurations, achieving validation macro-F1 of 0.9566 and MCC of 0.9160 at the default threshold. Under approximate FAR-target calibration at 0.01, it achieved validation macro-F1 of 0.9585, MCC of 0.9195, FAR of 0.0101, FNR of 0.0040, and attack recall of 0.9960. Because FAR is computed on a finite validation set, the calibrated operating point is reported as approximately target-FAR constrained rather than as an exact continuous FAR bound.

The optimized XGBoost hyperparameter configuration is provided in Supplementary Table S2.

### 3.7. Evaluation Protocols

To evaluate both in-distribution performance and generalization to unseen malicious behaviors, this study adopted four complementary protocols: closed-world 10-fold cross-validation, unseen attack family evaluation, repeated unseen-family evaluation, and threshold-calibrated operational evaluation.

#### 3.7.1. Closed-World 10-Fold Cross-Validation

The first protocol followed the conventional closed-world setting. The binary-labeled dataset was partitioned into ten folds using stratified sampling. In each run, nine folds were used for training and one fold was used for testing, and the process was repeated until every fold had served as the test partition once. Because the folds were stratified with respect to the binary label, the benign-to-attack proportion was preserved across all splits. This protocol measures in-distribution performance when the training and testing data are drawn from the same overall distribution.

The closed-world setting provides a stable benchmark for comparing model discrimination capability under standard experimental practice. However, since training and testing folds share the same family-level distribution, this protocol alone is insufficient to characterize robustness against previously unseen attack families.

#### 3.7.2. Unseen Attack Family Evaluation

The second protocol was designed to approximate a zero-day-like condition within the same dataset. In this setting, one malicious attack family was held out entirely from training and used only for testing. The protocol was repeated across all nine malicious families: DDoS, DoS, Mirai, Spoofing, Recon, Web, BruteForce, BrowserHijacking, and Backdoor.

For each held-out family, the test set consisted of all samples from that unseen malicious family and a held-out benign test pool. The benign test samples were separated from training and validation samples, so FAR was computed on genuine benign samples that were not seen during fitting or threshold calibration. The resulting family-level scores were averaged across all nine malicious families to obtain a model-level estimate of unseen-family generalization.

**Figure 2:**
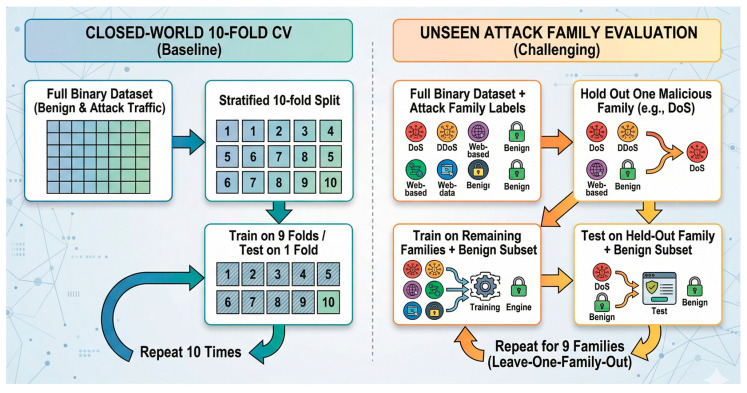
Evaluation design used in this study, including closed-world 10-fold cross-validation, unseen attack family evaluation, repeated sampling, and validation-based threshold calibration.

This protocol is more demanding than closed-world cross-validation because the model cannot rely on direct exposure to the held-out family during training. However, it should be interpreted as unseen-family or zero-day-like evaluation rather than a full open-world zero-day benchmark, because all samples still come from the same dataset and feature space. The evaluation design is illustrated in Figure 2.

#### 3.7.3. Repeated Unseen-Family Evaluation

To assess sampling variance, the unseen-family protocol was repeated over five random seeds. Each seed generated a benign split, a stratified 2,000,000-sample training budget, and a fitting/validation split. Because nine malicious families were evaluated under each seed, the repeated supplementary evaluation consisted of 45 unseen-family runs. Results are reported as mean ± standard deviation across these runs.

#### 3.7.4. Threshold-Calibrated and Cost-Sensitive Evaluation

The main experiments used the default threshold τ=0.5. However, because operational IDS deployment often requires explicit false alarm control, supplementary threshold-calibrated evaluation was also conducted. For each model and repeated unseen-family run, a decision threshold was selected on the validation set to approximate a target FAR. The evaluated FAR targets were 0.001, 0.005, 0.01, 0.02, and 0.05. The selected threshold was then applied to the unseen-family test set, and macro-F1, MCC, FAR, FNR, and attack recall were computed.

In addition, cost-sensitive threshold selection was performed by minimizing validation cost:

$$\mathrm{Cost}=C_{FP}\times FP+C_{FN}\times FN$$

where $C_{FP}$ is the cost assigned to a false positive and $C_{FN}$ is the cost assigned to a false negative. This analysis was used to examine how model preference changes when deployments prioritize benign alert suppression or missed-attack reduction.

### 3.8. Evaluation Metrics

Given the severe class imbalance in CICIoT2023, this study did not rely solely on accuracy. Instead, multiple complementary metrics were used to capture detection effectiveness, class balance, ranking quality, and false alarm behavior. Let TP, TN, FP, and FN denote true positives, true negatives, false positives, and false negatives, respectively.

Accuracy measures the proportion of correctly classified samples:

$$\mathrm{Accuracy}=\frac{TP+TN}{TP+TN+FP+FN}$$

Precision measures the proportion of predicted attack samples that are truly malicious:

$$\mathrm{Precision}=\frac{TP}{TP+FP}$$

Recall, also called attack recall in this binary setting, measures the proportion of attack samples correctly detected:

$$\mathrm{Recall}=\frac{TP}{TP+FN}$$

F1-score is the harmonic mean of precision and recall:

$$F1\mathrm{-score}=\frac{2\times \mathrm{Precision}\times \mathrm{Recall}}{\mathrm{Precision}+\mathrm{Recall}}$$

Macro-F1 was emphasized because it gives equal weight to both classes and is more informative under class imbalance than accuracy. Balanced accuracy was computed as the average of benign recall and attack recall:

$$\mathrm{Balanced}\;\mathrm{Accuracy}=\frac{TPR+TNR}{2}$$

where

$$TPR=\frac{TP}{TP+FN}$$

$$TNR=\frac{TN}{TN+FP}$$

MC was included because it provides a robust summary of binary classification quality under class imbalance:

$$MCC=\frac{TP\times TN-FP\times FN}{\sqrt{\left(TP+FP)\left(TP+FN\right)\left(TN+FP\right)(TN+FN\right)}}$$

False Alarm Rate (FAR) was defined as

$$FAR=\frac{FP}{FP+TN}$$

False Negative Rate (FNR) was defined as

$$FNR=\frac{FN}{FN+TP}$$

Attack recall is equivalent to

Attack Recall=1−FNR

ROC-AUC and PR-AUC were also reported as ranking metrics, but they were de-emphasized in the revised narrative because accuracy and attack-positive PR-AUC can be close to saturation under the extreme attack-dominant distribution.

For the closed-world setting, metrics were computed using out-of-fold predictions aggregated across the 10 folds. For unseen-family evaluation, metrics were first computed for each held-out family and then averaged across the nine malicious families. For repeated supplementary evaluation, results were summarized as mean ± standard deviation across 45 runs.

The notation and metric glossary is provided in Supplementary Table S3.

### 3.9. Explainability Analysis

To improve transparency and support interpretation of model behavior, this study incorporated feature contribution analysis into the proposed framework. Because the installed XGBoost runtime and the explainability stack were not fully compatible with the standard SHAP TreeExplainer path, the final experiments employed XGBoost native pred_contribs. This method produces SHAP-compatible feature contribution values in the model margin space. Accordingly, the explainability component of this study is described as XGBoost-native SHAP-compatible feature contribution analysis.

The explainability analysis was conducted at two levels. First, a global analysis was performed by computing the mean absolute contribution of each feature across the explanation set. This allowed the study to identify traffic features that most strongly influenced model decisions overall. Second, a local case analysis was conducted for four representative instances: a true positive, a false positive, a false negative, and a true negative. These cases reveal not only why the model succeeded on malicious and benign samples, but also why it produced a false alarm and why it failed to detect an attack instance.

To improve readability, local waterfall plots were supplemented with local top-contribution tables. In addition, a local reconstruction check was performed to clarify the behavior of native pred_contribs. The base margin plus the sum of feature contributions was compared with the model margin, and the corresponding sigmoid-transformed probability was compared with the model probability. This check confirmed negligible numerical reconstruction error. However, these values should be interpreted as margin-space contributions rather than probability-space SHAP values, because the sigmoid transformation is nonlinear.

The explainability stage therefore serves two purposes. First, it reveals which temporal, TCP flag-related, and packet-statistical features dominate the detection process. Second, it connects model performance to specific success and failure modes, thereby improving the practical interpretability of the proposed IoT attack detection framework.

The explainability analysis in this study should be interpreted as model-centered diagnostic interpretation rather than a fully analyst-centered explanation system. XGBoost native pred_contribs identifies which traffic features contributed to a model decision, but it does not by itself determine whether the resulting explanation is actionable for a security analyst. To move from feature attribution toward analyst-centered explanation, additional components would be required, including analyst-oriented alert summaries, traffic-context reconstruction, comparison with historical benign and malicious patterns, explanation confidence indicators, and triage-oriented recommendations. Therefore, the present work uses feature contribution analysis to support model inspection and error diagnosis, while leaving full analyst-centered explanation design and evaluation as a future research direction.

## 4. Experimental Results

### 4.1. Fair-Budget Closed-World Results

This section first reports the results of the fair-budget closed-world experiment. In this setting, all seven candidate models were trained under the same training budget of 2,000,000 samples and evaluated through stratified 10-fold cross-validation. The purpose of this design was to ensure that the comparison among models was not implicitly influenced by unequal training data exposure. This is particularly important in IoT intrusion detection, where large-scale tabular traffic data can favor models that benefit more directly from increased sample availability [2,4].

Under the fair-budget closed-world setting, tree-based ensemble methods clearly dominated the linear and shallow neural baselines. Random Forest achieved the highest macro-F1 of 0.9713, followed by LightGBM at 0.9602 and optimized XGBoost at 0.9566. Default XGBoost and CatBoost formed a second tier, with macro-F1 values of 0.9449 and 0.9444, respectively. The simple multilayer perceptron reached 0.9047, whereas Logistic Regression achieved only 0.7431. This overall ranking indicates that structured tree-based learners are particularly well matched to the statistical characteristics of CICIoT2023, which is consistent with the broader literature showing that boosting and ensemble tree models remain highly effective for tabular intrusion detection tasks [2,5,6].

From a conventional benchmark perspective, the closed-world results may appear to suggest that the attack detection problem is nearly saturated, especially for the strongest ensemble models. However, such an interpretation must be treated cautiously. High random-split or cross-validation performance does not necessarily imply robustness under evolving or unfamiliar malicious traffic conditions. Therefore, the closed-world experiment in this study should be interpreted primarily as a controlled in-distribution benchmark rather than as definitive evidence of real-world generalization.

Although Random Forest achieved the highest macro-F1 and MCC, its operational profile was less favorable when false alarms were considered. Specifically, Random Forest produced an FAR of 0.0499, which was substantially larger than those of LightGBM (0.0050), optimized XGBoost (0.0067), default XGBoost (0.0018), and CatBoost (0.0019). This result shows that superior macro-F1 under closed-world conditions does not automatically correspond to a preferable detector in practice. In an operational IoT environment, a model that generates excessive benign alerts may impose a significant cost on analysts and monitoring infrastructure.

Among the non-Random-Forest models, LightGBM and optimized XGBoost were the most competitive. LightGBM achieved the second-best macro-F1 and MCC while maintaining a far lower FAR than Random Forest. Optimized XGBoost also outperformed default XGBoost and CatBoost under macro-F1 and MCC while maintaining strong ranking quality. These results suggest that gradient-boosted tree models offer an effective balance between discrimination capability and operational conservativeness under fair-budget closed-world conditions.

**Figure 3:**
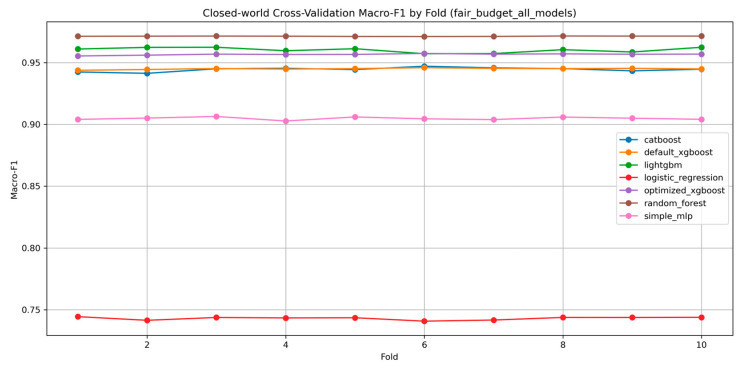
Fold-wise macro-F1 distribution of the fair-budget closed-world experiment. The figure illustrates the stability of each model across stratified folds under the same 2,000,000-sample training budget.

The fold-wise results also showed high stability. Random Forest exhibited the smallest variance across folds, and optimized XGBoost likewise maintained a highly stable fold-to-fold pattern. LightGBM, while strong overall, displayed slightly larger variability, though still within a narrow range. In contrast, Logistic Regression and simple MLP remained consistently weaker, further confirming that simple linear decision boundaries or lightweight neural structures are insufficient to match the strongest tree-based methods on this large-scale IoT benchmark. The fair-budget closed-world performance comparison is reported in Table 6. Fold-wise stability is illustrated in Figure 3 and summarized in Table 7.

### 4.2. Fair-Budget Unseen Attack Family Results

To evaluate robustness beyond in-distribution testing, all models were further assessed using the unseen attack family protocol. In this setting, one malicious family was entirely excluded from training and used only for testing. The reported results are the mean values over all nine held-out malicious families. This protocol provides a more stringent and operationally meaningful estimate of robustness than standard cross-validation, as it requires the detector to classify malicious traffic from a family not explicitly represented during training.

Under this unseen-family setting, the relative ranking among models changed in important ways. Random Forest still achieved the highest mean macro-F1 at 0.8433 and the lowest FNR at 0.0712, indicating the strongest recall-oriented detection among the compared models. However, it also produced an FAR of 0.0536, which remained substantially higher than those of all competing models. Optimized XGBoost and LightGBM emerged as the strongest lower-FAR alternatives, with mean macro-F1 values of 0.8194 and 0.8158, respectively. Their FAR values were 0.0086 and 0.0071, respectively, much lower than that of Random Forest. The mean unseen-family performance comparison is summarized in Table 8.

**Figure 4:**
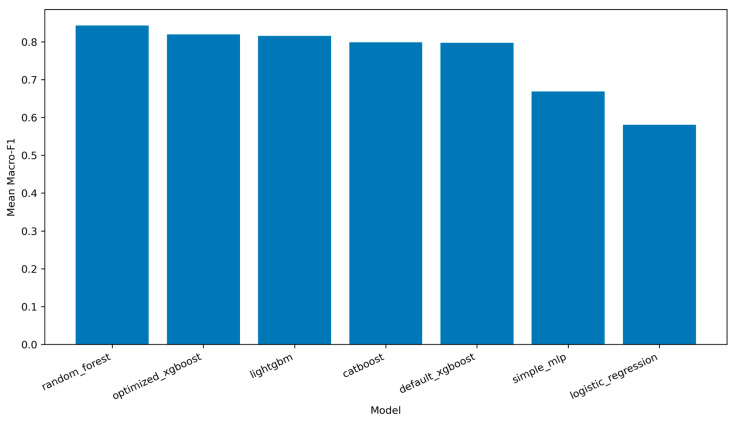
Mean macro-F1 of the fair-budget unseen attack family evaluation. The figure compares model robustness when one malicious family is completely excluded from training.

Figure 4 visualizes the mean macro-F1 values under the unseen attack family evaluation.

These results show that the best model depends on the deployment objective. If the objective is to minimize missed attacks, Random Forest is highly attractive because it produces the lowest FNR. If the objective is to reduce benign alert noise at the default operating threshold, optimized XGBoost and LightGBM provide more conservative operating points. This distinction is important because the dataset is extremely imbalanced and the FAR is computed only over genuine benign samples. In this evaluation, each unseen-family run included 219,639 held-out benign test samples. Therefore, even a seemingly moderate FAR can correspond to a nontrivial number of false alerts in large-scale monitoring.

The comparison between default XGBoost and optimized XGBoost demonstrates the value of the optimized configuration under the default threshold. In the unseen-family setting, optimized XGBoost improved the mean macro-F1 from 0.7973 to 0.8194 and increased the mean MCC from 0.6884 to 0.7067. However, because Random Forest retained a substantially lower FNR, the revised interpretation is not that optimized XGBoost universally dominates all baselines. Rather, optimized XGBoost is retained as the primary model of the proposed explainable XGBoost-based framework because it provides a competitive low-FAR default operating point, scales effectively in full-scale training, and directly supports native contribution-based explanation.

The family-wise analysis provides additional insight. DDoS, DoS, and Mirai remained relatively easy for all strong tree-based models, with macro-F1 values close to 1.0. In contrast, Spoofing, Recon, BruteForce, and some Web-related behaviors were markedly harder. This pattern suggests that large-scale volumetric or statistically distinctive attacks are easier to generalize across, whereas more subtle or heterogeneous attack families remain difficult when completely excluded from training. This observation is consistent with the XAI results, which show that the selected detector relies strongly on timing, TCP control-flag, and packet-statistical features. The family-wise macro-F1 values of selected models are reported in Table 9.

### 4.3. Repeated Sampling and Split Audit

To address sampling variance and reproducibility, the unseen-family evaluation was repeated over five random seeds. For each seed, 20% of benign traffic was held out as the benign test pool and combined with each held-out malicious family. The remaining benign samples and non-held-out malicious families formed the training candidate pool. From this pool, 2,000,000 samples were drawn by stratified sampling without replacement and split into 1,700,000 fitting samples and 300,000 validation samples. This process produced 45 repeated unseen-family runs.

The repeated default-threshold results were consistent with the original fair-budget unseen-family findings. Random Forest achieved the highest mean macro-F1 of 0.8415 ± 0.1182 and the highest attack recall of 0.9288, corresponding to the lowest FNR of 0.0712 ± 0.0930. However, it also produced the highest FAR of 0.0551 ± 0.0116. Optimized XGBoost achieved macro-F1 of 0.8196 ± 0.1812, MCC of 0.7075 ± 0.2425, FAR of 0.0090 ± 0.0026, and FNR of 0.2976 ± 0.2697. LightGBM obtained a similar profile, with macro-F1 of 0.8174 ± 0.1690, FAR of 0.0077 ± 0.0017, and FNR of 0.3151 ± 0.2862.

These repeated results confirm that the main conclusion is stable: Random Forest provides stronger recall-oriented detection, whereas optimized XGBoost and LightGBM provide lower-FAR default operating points. The large standard deviations in macro-F1 and FNR also indicate that unseen-family performance varies considerably by held-out family, reinforcing the importance of reporting family-aware and repeated evaluations rather than relying on a single split.

The split-overlap audit confirmed that no sample overlap existed between training and testing partitions. Across all 45 repeated runs, train–test overlap, fit–validation overlap, fit–test overlap, and validation–test overlap were all zero. This confirms that the repeated unseen-family evaluation was not affected by sample leakage.

The repeated default-threshold unseen-family results are provided in Supplementary Table S4.

### 4.4. Threshold-Calibrated Evaluation Under Fixed-FAR Constraints

**Figure 5:**
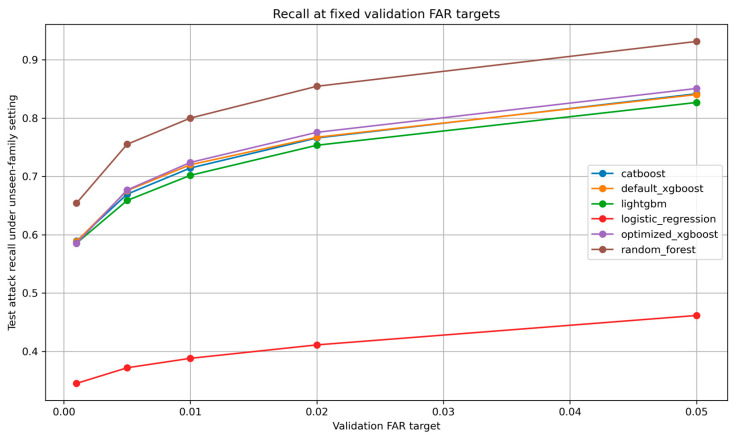
Attack recall under validation-based fixed-FAR threshold calibration. The figure shows that attack recall improves as the FAR target is relaxed, while stricter FAR targets substantially increase missed detections for all models.

Because operational deployment often requires explicit false-alarm control, validation-based threshold calibration was performed under fixed-FAR targets. For each repeated unseen-family run, the threshold was selected on the validation set to approximate a target FAR and then evaluated on the held-out unseen-family test set. This experiment directly addresses whether model ranking changes when models are compared at similar false-alarm operating points rather than only at the default threshold. Figure 5 illustrates attack recall under different validation-based fixed-FAR targets.

At the approximate FAR target of 0.01, Random Forest achieved the strongest calibrated recall-oriented performance. It obtained macro-F1 of 0.8754 ± 0.0962, MCC of 0.7757, observed FAR of 0.0102 ± 0.0012, FNR of 0.2000 ± 0.1580, and attack recall of 0.8000. Optimized XGBoost remained competitive but did not dominate under the calibrated setting, achieving macro-F1 of 0.8323 ± 0.1603, MCC of 0.7193, observed FAR of 0.0105 ± 0.0013, FNR of 0.2760 ± 0.2445, and attack recall of 0.7240. Default XGBoost, CatBoost, and LightGBM produced similar calibrated profiles, while Logistic Regression remained clearly weaker.

This result changes the interpretation of the model ranking. Under the default threshold, optimized XGBoost provides a favorable low-FAR operating point compared with Random Forest. However, when all models are calibrated to an approximately equal FAR target, Random Forest achieves substantially better attack recall and lower FNR. Therefore, the revised conclusion is deployment-objective dependent: Random Forest is preferable when the operational objective is to maximize detection under a fixed-FAR constraint, whereas optimized XGBoost remains a strong primary model for an explainable, low-FAR XGBoost-based framework emphasizing scalability and native interpretability.

The validation-based threshold calibration results are provided in Supplementary Table S5.

The fixed-FAR analysis also demonstrates why reporting only the default threshold can be incomplete. At FAR target = 0.001, all models became much more conservative and suffered higher FNR. As the FAR target increased from 0.001 to 0.05, attack recall improved for all models, but the number of false alarms also increased. This confirms that false alarm control and missed-attack reduction are inherently competing objectives.

### 4.5. Cost-Sensitive Threshold Analysis

To further examine deployment-dependent model selection, cost-sensitive threshold analysis was performed by minimizing a validation-set cost function that assigns different weights to false positives and false negatives. This analysis is important because different IoT security deployments may have different operational priorities. A highly constrained monitoring team may assign a larger cost to false positives to avoid alert fatigue, while a high-risk security environment may assign a larger cost to false negatives to avoid missed attacks.

When FP and FN were assigned equal cost, Random Forest remained strong, achieving macro-F1 of 0.8457, MCC of 0.7320, FAR of 0.0493, FNR of 0.0840, and attack recall of 0.9160. Optimized XGBoost achieved macro-F1 of 0.8017, MCC of 0.6700, FAR of 0.0515, FNR of 0.1614, and attack recall of 0.8386 under the same equal-cost setting. When false negatives were assigned a higher cost, thresholds became more aggressive, attack recall increased, and FAR also increased. Conversely, when false positives were assigned a higher cost, thresholds became more conservative and FAR decreased, but FNR increased.

These findings reinforce the main operational interpretation of this study. Model selection should not be based on a single aggregate metric. Instead, it should be based on deployment risk tolerance, acceptable false alarm budget, and the relative cost of missed attacks. Optimized XGBoost remains valuable as a low-FAR and explainable XGBoost-based detector, whereas Random Forest is more suitable when the primary cost is missing attacks.

### 4.6. Supplementary Full-Scale Optimized XGBoost Results

To complement the fair-budget comparison, a supplementary experiment was conducted using optimized XGBoost trained without the shared 2,000,000-sample constraint. The purpose of this experiment was not to replace the fair-budget comparison but to determine whether the selected primary model would further benefit from full-scale training on the entire dataset.

In the closed-world setting, full-scale optimized XGBoost achieved an accuracy of 0.9956, a balanced accuracy of 0.9969, a macro-F1 of 0.9560, and an MCC of 0.9155. These values were very close to those of the fair-budget optimized XGBoost setting. However, the FAR decreased substantially from 0.0067 to 0.0017. This suggests that under closed-world conditions, increasing the training scale did not dramatically improve already high in-distribution discrimination, but it did improve the model’s conservativeness with respect to benign traffic.

The full-scale setting became more informative under unseen-family evaluation. Compared with fair-budget optimized XGBoost, the full-scale version improved the mean macro-F1 from 0.8194 to 0.8300 and increased the mean MCC from 0.7067 to 0.7344. Its mean PR-AUC also improved from 0.8858 to 0.9178, while the mean FAR decreased from 0.0086 to 0.0021. This result indicates that optimized XGBoost scales effectively with larger training data volumes, especially when low false alarm behavior is prioritized.

At the same time, not all metrics improved uniformly. The full-scale model exhibited a slightly lower mean balanced accuracy and a somewhat higher FNR than the fair-budget version. This suggests that the performance improvement was not due to a uniform increase in sensitivity across all difficult families but rather to a shift in decision behavior that particularly reduced benign misclassification and improved ranking quality. In other words, full-scale training made the model more conservative toward benign traffic, which reduced FAR and improved MCC and PR-AUC but also increased missed detections for some difficult unseen-family attack samples.

Taken together, these supplementary results show that optimized XGBoost benefits from larger-scale training, especially in terms of FAR, MCC, PR-AUC, and macro-F1 under unseen-family evaluation. However, the full-scale results should not be interpreted as a uniform improvement across all metrics. Full-scale optimized XGBoost is best interpreted as a scalable low-FAR model rather than as a universally superior detector. It remains valuable as the primary model of the proposed explainable XGBoost-based framework because it combines strong default-threshold performance, very low FAR, full-scale scalability, and native contribution-based interpretability. Nevertheless, the threshold-calibrated analysis shows that Random Forest should be preferred when the deployment objective is to maximize attack recall under a fixed-FAR constraint. The comparison between fair-budget and full-scale optimized XGBoost is summarized in Table 10.

### 4.7. XAI-Based Global and Local Interpretation

To better understand the behavior of the selected optimized XGBoost model, explainability analysis was performed using XGBoost native pred_contribs, which produces SHAP-compatible contribution values in the model margin space. This design provides both global and local interpretability while remaining consistent with the software environment used in the experiments.

**Figure 6:**
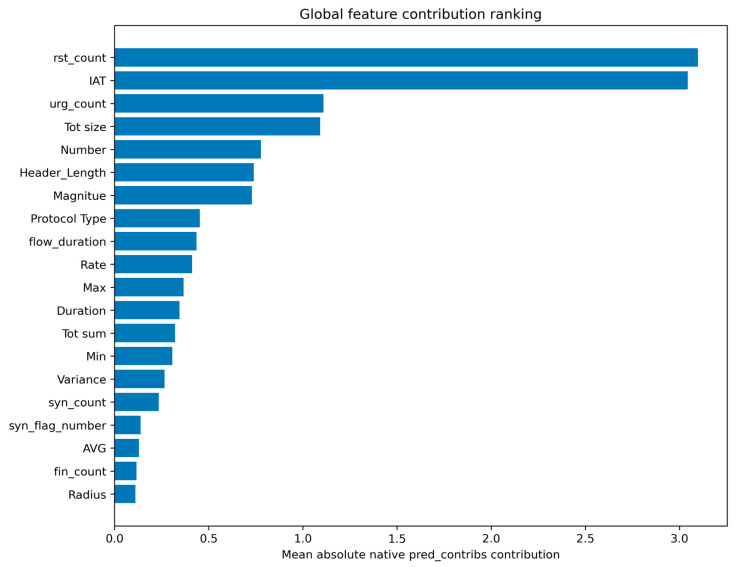
Global feature contribution ranking of the selected optimized XGBoost model using XGBoost native pred_contribs in margin space. The ranking indicates that TCP control-flag, temporal, and packet-statistical features dominate the model’s malicious-versus-benign decisions.

At the global level, the revised analysis showed that the most influential features were rst_count, IAT, urg_count, Tot size, Number, Header_Length, Magnitude, Protocol Type, flow_duration, and Rate. These variables can be grouped into three broad categories: TCP control-flag behavior, temporal behavior, and packet or flow statistical descriptors. The dominance of rst_count and urg_count indicates that abnormal TCP control behavior contributes strongly to the malicious decision boundary. The importance of IAT and flow_duration suggests that timing irregularity and communication pacing are central to the detector. The contributions of Tot size, Number, Header_Length, Magnitude, and Rate further indicate that the model captures traffic volume and packet-statistical characteristics. The global feature contribution ranking is shown in Figure 6. The top 10 global feature contributions are provided in Supplementary Table S6.

The local analysis was expanded from compressed waterfall plots to top-contribution summary tables for representative TP, FP, FN, and TN cases. This improves readability and makes it easier to identify which features pushed the decision toward attack or benign. In the representative TP case, rst_count, IAT, urg_count, Rate, Tot size, fin_count, Magnitude, flow_duration, Header_Length, and syn_flag_number all contributed positively toward the attack prediction. This indicates that the model recognized a strong combination of TCP control, timing, and packet-statistical evidence.

**Figure 7:**
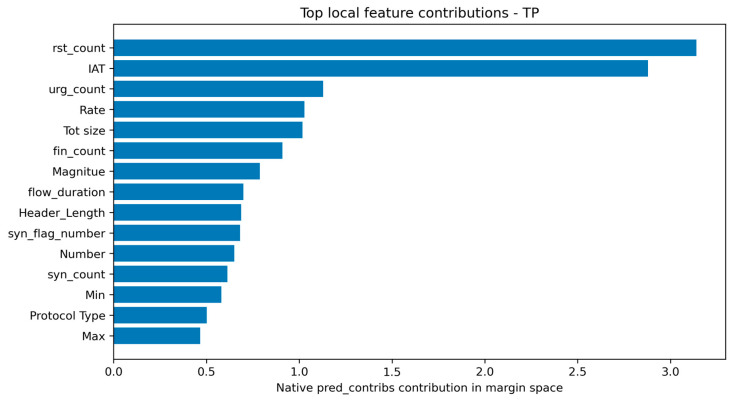
Local feature contribution analysis for a representative true-positive case. Positive contributions indicate traffic characteristics that push the model toward an attack prediction.

**Figure 8:**
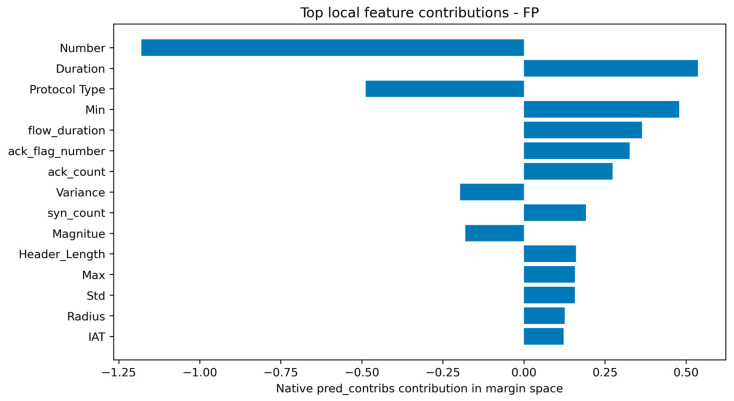
Local feature contribution analysis for a representative false-positive case. The case illustrates how benign traffic can partially resemble malicious traffic in the dominant timing, TCP control, and packet-statistical feature space.

The representative FP case is operationally important because it reveals why a benign sample was incorrectly classified as malicious. Its predicted score was close to the decision boundary, indicating a low-margin false alarm rather than an overwhelmingly confident attack decision. The contribution table suggests that some benign traffic patterns partially resembled malicious behavior in the same feature dimensions used for attack detection. Conversely, the representative FN case had a score close to the threshold but slightly below it, showing that some malicious samples can remain near the benign side of the learned boundary. These cases are particularly useful for identifying residual blind spots and for guiding future feature refinement. Representative true-positive and false-positive local explanations are shown in Figure 7 and Figure 8, respectively.

**Figure 9:**
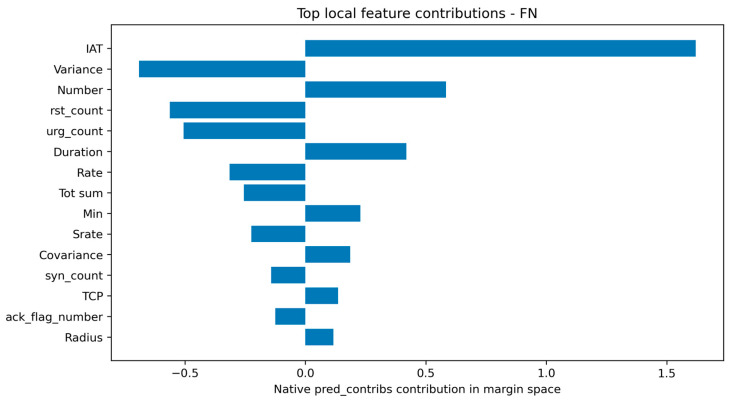
Local feature contribution analysis for a representative false-negative case. The case illustrates a missed attack sample whose feature pattern remains close to the benign side of the learned decision boundary.

**Figure 10:**
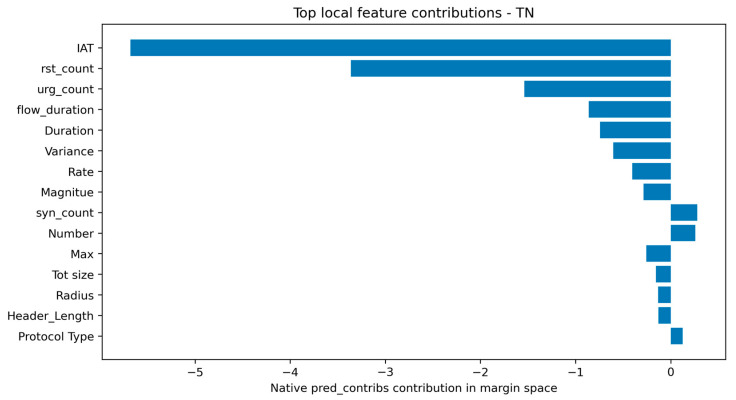
Local feature contribution analysis for a representative true-negative case. The case illustrates benign-side evidence that supports a correct benign prediction.

To clarify the interpretation of native pred_contribs, a local reconstruction check was performed. For the selected cases, the base margin plus the sum of feature contributions reconstructed the model margin, and the sigmoid transformation of this reconstructed margin reproduced the model probability with negligible numerical error. The absolute probability gaps were approximately 4.35 × 10^−8^ for the TP case, 1.71 × 10^−7^ for the FP case, 6.71 × 10^−8^ for the FN case, and 4.96 × 10^−12^ for the TN case. This confirms the local additive reconstruction property in margin space. However, because the sigmoid transformation is nonlinear, the reported contributions should be interpreted as margin-space contributions rather than probability-space SHAP values. Representative false-negative and true-negative local explanations are shown in Figure 9 and Figure 10, respectively. The local reconstruction check is provided in Supplementary Table S7.

Overall, the XAI results support the technical plausibility of the optimized XGBoost detector. The model relies on traffic features that are meaningful in intrusion detection, especially TCP control flags, timing behavior, and packet-statistical descriptors. At the same time, the local FP and FN cases show that feature attribution should be treated as diagnostic model evidence rather than as a complete human-centered explanation. Fully actionable analyst-centered explanation design remains an important direction for future work.

### 4.8. Summary of Experimental Findings

The experimental results support six main findings.

First, under fair-budget closed-world evaluation, tree-based ensemble models clearly outperformed the linear and shallow neural baselines, confirming the suitability of boosting and ensemble trees for large-scale IoT tabular traffic analysis.

Second, the shift from closed-world to unseen attack family evaluation revealed a substantial gap between in-distribution performance and unseen-family robustness. This supports the argument that standard benchmark settings alone are insufficient for assessing operationally meaningful intrusion detection performance.

Third, the repeated unseen-family results confirmed that model ranking depends strongly on the deployment objective. Random Forest achieved the strongest recall-oriented detection and lowest FNR, while optimized XGBoost and LightGBM provided substantially lower-FAR default operating points.

Fourth, validation-based fixed-FAR calibration changed the interpretation of model preference. At an approximate FAR target of 0.01, Random Forest achieved the best calibrated recall-oriented performance, whereas optimized XGBoost remained competitive but did not dominate. This shows that threshold calibration is necessary when making practical claims about false-alarm control.

Fifth, the supplementary full-scale optimized XGBoost experiment showed that larger-scale training improved macro-F1, MCC, PR-AUC, and FAR under unseen-family evaluation, but also slightly decreased balanced accuracy and increased FNR. This non-monotonic behavior indicates an operating-point shift toward stronger benign alert suppression rather than uniform improvement across all metrics.

Sixth, the XAI analysis showed that optimized XGBoost decisions were dominated by TCP control-flag, temporal, and packet-statistical features. Local TP, FP, FN, and TN contribution tables improved explanation readability and confirmed additive reconstruction of native pred_contribs in margin space.

Overall, the findings indicate that the proposed framework is useful not because optimized XGBoost strictly dominates every baseline, but because it provides a reproducible and deployment-aware evaluation framework. Optimized XGBoost is a strong explainable low-FAR model, while Random Forest is preferable when the primary objective is maximizing attack recall under a fixed-FAR constraint.

## 5. Discussion

### 5.1. Interpretation of Closed-World and Unseen-Family Results

The experimental results reveal a clear and meaningful gap between conventional closed-world performance and unseen attack family generalization. Under the fair-budget closed-world setting, several tree-based ensemble models achieved very high performance, with Random Forest, LightGBM, and optimized XGBoost all producing macro-F1 values above 0.95. From a purely benchmark-oriented perspective, such results might suggest that the binary IoT attack detection problem is nearly saturated on CICIoT2023. However, the unseen-family evaluation demonstrates that this interpretation would be incomplete.

Once the protocol was changed so that an entire malicious family was excluded from training, all models experienced a noticeable decline in macro-F1 and related metrics. This result highlights the difference between in-distribution discrimination and generalization to unfamiliar malicious behaviors. In standard stratified cross-validation, the model benefits from the fact that training and test folds are drawn from the same overall distribution. Even if the samples are not identical, they often share family-level statistical regularities. By contrast, the unseen-family protocol forces the detector to identify malicious traffic from an attack family that was not directly represented during training. This makes the problem more aligned with the uncertainty that intrusion detection systems face after deployment.

The revised experiments further show that unseen-family performance is sensitive to family identity and sampling variation. DDoS, DoS, and Mirai remained comparatively easy for the stronger tree-based models, while Spoofing, Recon, BruteForce, and some Web-related traffic were substantially more difficult. This suggests that attack families characterized by stronger volumetric, temporal, or distributional signatures are easier to recognize even when the exact family is absent during training. Conversely, families whose behavior is subtler, less voluminous, or more heterogeneous appear to overlap more strongly with benign traffic in feature space.

This interpretation is consistent with the XAI results. The optimized XGBoost model relied heavily on TCP control-flag behavior, timing behavior, and packet-statistical descriptors. These signals are likely to be more distinctive for large-scale or highly patterned attacks than for low-intensity or behaviorally mixed families. Therefore, unseen-family evaluation should be viewed not as an optional secondary experiment but as an important complement to standard benchmark testing.

### 5.2. Deployment-Objective-Dependent Model Selection

One of the most important findings of this study is that the best-performing model depends on the deployment objective. If the goal is simply to maximize macro-F1 or attack recall under the unseen-family setting, Random Forest appears highly attractive. It achieved the highest macro-F1 and the lowest FNR in both the original fair-budget unseen-family experiment and the repeated supplementary evaluation. In the repeated default-threshold setting, Random Forest achieved macro-F1 of 0.8415 and FNR of 0.0712, meaning that it missed far fewer attacks than optimized XGBoost.

However, this recall-oriented advantage came with a much higher false alarm rate. Random Forest produced an FAR of approximately 0.0551 in the repeated default-threshold unseen-family evaluation, whereas optimized XGBoost produced an FAR of approximately 0.0090. Because each repeated unseen-family run included 219,639 genuine benign test samples, this difference is operationally meaningful. A 5% FAR can generate a large number of benign alerts in high-volume IoT monitoring, increasing analyst burden and reducing trust in the detector.

The fixed-FAR calibration experiment adds a more nuanced view. When models were calibrated to an approximate FAR target of 0.01, Random Forest achieved the strongest calibrated recall-oriented performance, with macro-F1 of 0.8754, MCC of 0.7757, FNR of 0.2000, and attack recall of 0.8000. Optimized XGBoost remained competitive, with macro-F1 of 0.8323, MCC of 0.7193, FNR of 0.2760, and attack recall of 0.7240, but it did not outperform Random Forest at this calibrated operating point.

This result directly supports a deployment-objective-dependent interpretation. If an organization prioritizes minimizing missed attacks under a fixed-FAR constraint, Random Forest may be the preferred detector. If an organization prioritizes a low-FAR default operating point, scalability, and native explainability compatibility within an XGBoost-based framework, optimized XGBoost remains a strong primary model. Therefore, the revised conclusion is not that optimized XGBoost universally dominates all baselines, but that it provides a favorable and explainable low-FAR operating profile under the proposed framework.

### 5.3. Threshold Calibration and Cost-Sensitive Implications

The threshold-calibrated results demonstrate why fixed-threshold evaluation is insufficient for deployment-oriented IDS comparison. At the default threshold, model outputs reflect not only ranking quality but also model-specific calibration behavior. A model may appear overly aggressive or overly conservative simply because its raw probability scores are distributed differently from those of another model. Therefore, comparing all models only at τ = 0.5. can lead to incomplete operational conclusions.

Validation-based fixed-FAR calibration addresses this issue by selecting a threshold that approximates a specified false alarm constraint. This makes the comparison more meaningful for operational environments where alert budgets are limited. The results show that model ranking changes after calibration: Random Forest, which had the highest default FAR, became the strongest recall-oriented model under the approximate FAR target of 0.01. This finding confirms that false-alarm control should not be inferred only from default-threshold results.

Cost-sensitive threshold analysis further reinforces the same point. When the cost of false positives is high, conservative models or higher thresholds become more attractive because they suppress benign alerts. When the cost of false negatives is high, lower thresholds or recall-oriented models become preferable because missing attacks is more expensive than investigating benign alerts. Thus, the “optimal” model cannot be determined by a single aggregate metric alone. It should be selected according to deployment risk tolerance, alert handling capacity, and the relative operational costs of false positives and false negatives.

### 5.4. Interpretation of Full-Scale Optimized XGBoost Results

The supplementary full-scale optimized XGBoost experiment showed both improvements and non-monotonic behavior. Compared with fair-budget optimized XGBoost under unseen-family evaluation, the full-scale model improved macro-F1 from 0.8194 to 0.8300, increased MCC from 0.7067 to 0.7344, improved PR-AUC from 0.8858 to 0.9178, and reduced FAR from 0.0086 to 0.0021. These results indicate that larger-scale training helped the model improve overall ranking quality and suppress benign false alarms.

However, balanced accuracy decreased from 0.8459 to 0.8323, and FNR increased from 0.2996 to 0.3334. This behavior shows that the improvement was not uniform across all metrics. The most plausible interpretation is an operating-point shift. Full-scale training made the model more conservative with respect to benign traffic, reducing false alarms and improving MCC and macro-F1 through better benign handling. At the same time, this conservativeness increased missed detections for some difficult unseen-family attack samples, resulting in higher FNR and lower balanced accuracy.

This non-monotonic behavior is not contradictory. Macro-F1, MCC, FAR, FNR, balanced accuracy, ROC-AUC, and PR-AUC capture different aspects of model behavior. A model can improve false alarm control and ranking quality while losing some sensitivity to difficult attacks at a fixed threshold. Therefore, full-scale optimized XGBoost should be interpreted as a scalable low-FAR detector rather than as a uniformly better detector across all operational objectives.

### 5.5. Implications of the XAI Findings

The XAI analysis provides important insight into how the selected optimized XGBoost model makes decisions and why certain attack families are easier to detect than others. At the global level, the most influential features were rst_count, IAT, urg_count, Tot size, Number, Header_Length, Magnitude, Protocol Type, flow_duration, and Rate. These variables can be grouped into three broad categories: TCP control-flag behavior, temporal behavior, and packet or flow statistical descriptors.

The importance of rst_count and urg_count suggests that abnormal TCP control behavior contributes strongly to the malicious decision boundary. The importance of IAT and flow_duration indicates that timing irregularity and communication pacing are central to the model’s discrimination logic. The contribution of Tot size, Number, Header_Length, Magnitude, and Rate further indicates that the model captures traffic volume and packet-statistical patterns.

These observations help explain the differential performance across unseen families. Families such as DDoS, DoS, and Mirai likely produced strong deviations in timing, traffic size, and repetitive flow behavior, making them more separable even when they were not directly observed during training. In contrast, Spoofing, Recon, BruteForce, and some Web-related attacks likely generated patterns that were more subtle or partially overlapped with benign traffic in these feature dimensions.

The revised local explanation analysis also improves interpretability. Instead of relying only on compressed waterfall plots, local contribution tables show the top features that push each representative case toward attack or benign. The TP case demonstrates how strong TCP, timing, and traffic-size evidence support a confident attack prediction. The FP case shows how benign traffic can partially resemble malicious behavior. The FN case reveals that some malicious samples remain close to the benign side of the decision boundary. The TN case provides a complementary example of strong benign-side evidence.

It is important to interpret native pred_contribs correctly. The reconstruction check confirms that the base margin plus feature contributions reconstructs the model margin and reproduces the predicted probability with negligible numerical error after sigmoid transformation. However, these contributions are margin-space explanations, not probability-space SHAP values. Therefore, they should be treated as diagnostic model evidence rather than as complete causal or human-centered explanations.

### 5.6. Toward Actionable Analyst-Centered Explanations

The explainability component of this study provides global and local feature contribution evidence for the optimized XGBoost detector. These explanations help identify which traffic characteristics influence model predictions and why representative true-positive, false-positive, false-negative, and true-negative cases occur. However, feature attribution alone is not equivalent to an actionable analyst-centered explanation. In practical security operations, analysts need explanations that support triage, prioritization, investigation, and response decisions rather than only indicating which numerical features contributed to a model score.

To move toward actionable analyst-centered explanations, at least four additional components would be needed. First, the explanation output should be connected to an analyst-facing alert interface. Instead of presenting only feature contribution values, the system should translate the dominant features into a concise alert narrative, such as whether the decision was mainly driven by abnormal TCP control flags, unusual inter-arrival timing, high packet-rate behavior, or flow-size deviations. This interface should allow analysts to inspect the raw traffic evidence, compare the current event with similar historical alerts, and determine whether the model decision is operationally plausible.

Second, an analyst-centered explanation system should include explicit evaluation protocols. Possible evaluation criteria include explanation usefulness, explanation clarity, triage time, analyst confidence calibration, false-positive review efficiency, and decision consistency across analysts. These criteria cannot be fully assessed using offline classification metrics alone. They require user-centered experiments in which security analysts or trained participants review alerts with and without explanations and the resulting investigation outcomes are compared.

Third, explanation quality should be evaluated separately for different operational cases. True-positive explanations should help analysts understand why an alert is credible. False-positive explanations should help determine whether benign traffic resembles malicious behavior and whether threshold adjustment or allow-listing is appropriate. False-negative analysis should support blind-spot discovery by identifying malicious samples that remain close to the benign side of the decision boundary. True-negative explanations can help verify that the model correctly recognizes benign traffic patterns. Therefore, TP, FP, FN, and TN explanations should be evaluated not only visually but also according to their usefulness in realistic triage workflows.

Fourth, actionable explanations should be integrated with threshold and risk settings. Because this study shows that model preference changes under fixed-FAR and cost-sensitive objectives, an analyst-centered interface should also expose the operational threshold, expected false alarm trade-off, and confidence or margin of each decision. This would allow analysts and system administrators to understand how a given alert relates to the selected deployment objective, such as minimizing false alarms or minimizing missed attacks.

Accordingly, the present XAI analysis should be viewed as a necessary but not sufficient step toward operational explainability. It provides diagnostic model evidence and identifies meaningful traffic-level features, but future work is needed to transform these feature attributions into analyst-facing explanation workflows, human-subject evaluation protocols, and practical interfaces for IoT security operations. Table 11 summarizes the main requirements for moving from model-centered attribution to analyst-centered explanation.

### 5.7. Practical Significance for IoT Cybersecurity

The practical contribution of this study lies in demonstrating that a strong IoT attack detector should not be judged only by closed-world accuracy or a single F1-score. In real deployment settings, a detector must satisfy multiple requirements simultaneously: it must detect malicious traffic accurately, remain reasonably robust to unfamiliar malicious families, limit false alarms to manageable levels, provide enough interpretability to support analyst review, and allow threshold adjustment according to deployment constraints.

The additional discussion on analyst-centered explanation further clarifies that practical IoT cybersecurity deployment requires not only accurate and calibrated detection, but also explanation formats that reduce analyst workload and improve triage decisions.

The fair-budget comparison is practically important because it controls for unequal data access across models. The repeated unseen-family evaluation is practically important because it reveals sampling variance and family-dependent difficulty. The fixed-FAR calibration experiment is practically important because operational IDS deployments often face explicit alert budgets. The XAI analysis is practically important because analysts need to understand which traffic characteristics drove the model output.

From this perspective, optimized XGBoost is a strong candidate for an explainable low-FAR XGBoost-based framework, especially when native feature contribution analysis, scalability, and conservative alert behavior are important. However, Random Forest is a strong alternative when the primary deployment objective is maximizing attack recall under a fixed-FAR constraint. This distinction makes the revised framework more realistic: instead of presenting a universal winner, it provides a structured basis for selecting models according to operational priorities.

### 5.8. Limitations of This Study

Despite its contributions, this study has several limitations.

First, the experiments were conducted on a single benchmark dataset, CICIoT2023. Although CICIoT2023 is large, realistic, and well suited to IoT attack detection research, the conclusions may still be influenced by dataset-specific traffic composition, device diversity, and attack generation procedures. A model that performs strongly on CICIoT2023 may not generalize equally well to other IoT environments.

Second, the unseen attack family protocol should be interpreted as unseen-family or zero-day-like evaluation rather than a full open-world zero-day benchmark. Because all experiments were still conducted within a single dataset and feature space, some shared low-level traffic structure may remain across training and testing even when one malicious family is held out.

Third, the current formulation is binary rather than multiclass. While binary malicious-versus-benign detection is highly relevant for practical traffic screening, it does not directly address attack attribution after detection. In operational settings, distinguishing between attack categories may be important for response prioritization and forensic interpretation.

Fourth, the calibration experiments were validation-based and performed within the same benchmark environment. Real deployment would require ongoing calibration monitoring because benign traffic profiles and attack distributions may shift over time.

Fifth, the explainability component relied on XGBoost native pred_contribs rather than a fully harmonized comparison across multiple explanation backends. Although the local reconstruction check confirms additive reconstruction in margin space, future work should compare attribution stability across standard SHAP TreeExplainer, native pred_contribs, and other explanation methods.

Sixth, the study focuses mainly on model-centered explanations. While local contribution tables improve readability, they are not a substitute for fully user-centered explanation design. Future work should evaluate whether analysts find these explanations actionable, trustworthy, and useful for triage decisions.

Seventh, although this study adds global and local XAI analyses, it does not evaluate whether the explanations are actionable for human analysts. The current explanation outputs remain model-centered and are based on feature contributions in the model margin space. Future analyst-centered evaluation should examine whether these explanations improve triage accuracy, reduce investigation time, support threshold adjustment, and help analysts identify false positives and false negatives more effectively.

Overall, the revised discussion indicates that the proposed framework is most valuable not because one model dominates every metric, but because it provides a reproducible and deployment-aware basis for IoT attack detector evaluation. Table 12 summarizes the main strengths and limitations of the proposed study.

## 6. Conclusions

### 6.1. Concluding Remarks

This paper presented an explainable XGBoost-based framework for IoT attack detection with unseen attack family evaluation using CICIoT2023 as the large-scale experimental benchmark. The study was motivated by several methodological and practical issues that remain insufficiently addressed in much of the existing IoT intrusion detection literature: overreliance on closed-world evaluation, unclear fair-budget sampling strategies, limited reproducibility of optimized model configurations, fixed-threshold operational claims, and insufficient integration of explainability into diagnostic model assessment.

The experimental results demonstrated that tree-based ensemble models clearly outperform the linear and shallow neural baselines for large-scale IoT tabular traffic data. Under fair-budget closed-world cross-validation, Random Forest achieved the highest macro-F1, while LightGBM and optimized XGBoost also delivered strong and stable performance. However, the unseen attack family evaluation provided a more informative view of robustness. Random Forest achieved the strongest recall-oriented detection and the lowest FNR, whereas optimized XGBoost and LightGBM provided lower-FAR default operating points.

The repeated unseen-family evaluation confirmed that this trade-off is stable across random seeds. Random Forest achieved the highest repeated default-threshold macro-F1 and attack recall but also generated the highest false alarm rate. Optimized XGBoost achieved a substantially lower FAR while retaining competitive macro-F1 and MCC. These results show that the best model cannot be determined by a single aggregate metric alone.

The validation-based fixed-FAR calibration experiment further refined the conclusion. At an approximate FAR target of 0.01, Random Forest achieved the strongest calibrated recall-oriented performance, with macro-F1 of 0.8754, MCC of 0.7757, FNR of 0.2000, and attack recall of 0.8000. Optimized XGBoost remained competitive, with macro-F1 of 0.8323, MCC of 0.7193, FNR of 0.2760, and attack recall of 0.7240. This demonstrates that threshold calibration is essential when making operational claims about false alarm control.

The supplementary full-scale optimized XGBoost experiment showed that larger-scale training improved macro-F1, MCC, PR-AUC, and FAR under unseen-family evaluation, but also increased FNR and slightly reduced balanced accuracy. This non-monotonic behavior suggests that full-scale training shifted the model toward stronger benign alert suppression rather than uniformly improving attack sensitivity. Therefore, full-scale optimized XGBoost should be interpreted as a scalable low-FAR model rather than as a universally superior detector.

The explainability analysis further strengthened the value of the proposed framework. Using XGBoost-native SHAP-compatible feature contribution analysis, the study showed that model decisions were dominated by TCP control-flag behavior, temporal characteristics, and packet or flow statistical descriptors. The global ranking of features such as rst_count, IAT, urg_count, Tot size, Number, Header_Length, and Magnitude suggested that the model learned plausible traffic-level indicators of malicious behavior. Local contribution tables for representative TP, FP, FN, and TN cases provided interpretable insight into both successful detections and residual errors. The local reconstruction check confirmed that native pred_contribs reconstructs the model margin with negligible numerical error, while the study explicitly clarified that these values should be interpreted as margin-space contributions.

Overall, the main contribution of this study lies not simply in obtaining high detection scores, but in proposing a more rigorous, reproducible, explainable, and deployment-aware IoT intrusion detection evaluation framework. The results suggest that optimized XGBoost is a strong primary model for an explainable low-FAR XGBoost-based framework, especially when scalability and native contribution-based interpretation are required. However, Random Forest may be preferred when the deployment objective is to maximize attack recall under a fixed-FAR constraint. Thus, the optimal model choice is deployment-objective-dependent rather than universally determined by one metric.

### 6.2. Future Work

Several directions can be explored to extend this research.

First, future work should evaluate the proposed framework on additional IoT cybersecurity datasets and under cross-dataset transfer settings. Such experiments would help determine whether the observed trade-offs among Random Forest, optimized XGBoost, LightGBM, and other baselines persist across different device ecosystems, traffic characteristics, and attack-generation procedures.

Second, the current binary formulation could be extended to multiclass or hierarchical intrusion detection. In practical deployment, identifying traffic as malicious is only the first step; analysts may also need to know which attack family or attack category is most likely responsible. A two-stage or hierarchical framework that first performs malicious-versus-benign screening and then performs family attribution could improve incident triage and response planning.

Third, future studies should investigate more advanced calibration and cost-sensitive learning strategies. The present study added validation-based fixed-FAR threshold calibration and cost-sensitive threshold selection, but real IoT deployments may require dynamic threshold adjustment under changing traffic conditions. Online calibration, drift-aware thresholding, and alert-budget-aware optimization are promising extensions.

Fourth, additional representation learning and feature enhancement techniques could be explored for the more difficult unseen families, particularly Spoofing, Recon, BruteForce, and Web-related attacks. These families exhibited weaker generalization performance than DDoS, DoS, and Mirai, suggesting that their malicious behavior may not be fully captured by the current feature space. Future work may examine richer temporal modeling, family-aware representation learning, graph-based traffic modeling, or hybrid approaches that combine structured flow statistics with sequential context.

Fifth, the explainability component can be expanded further. Although the current study successfully employed XGBoost-native SHAP-compatible feature contribution analysis and verified local margin-space reconstruction, future work may compare multiple explanation backends under a fully harmonized software environment. Such comparison would help evaluate attribution stability and improve confidence in explanation consistency.

Sixth, future work should move from model-centered attribution toward actionable analyst-centered explanation design. Although the current study provides global feature rankings, local TP/FP/FN/TN contribution summaries, and margin-space reconstruction checks, these outputs do not yet constitute a complete operational explanation system. Future work should develop analyst-facing interfaces that translate feature contributions into concise alert narratives, show supporting traffic evidence, compare the event with similar historical cases, and expose the selected operating threshold and false-alarm trade-off. User studies with security analysts should also be conducted to evaluate whether the explanations improve triage accuracy, reduce investigation time, calibrate analyst trust, and support more consistent incident response decisions.

Finally, the present framework may be extended toward real-time or streaming IoT intrusion detection. Since practical IoT environments continuously generate traffic data, future research could investigate online learning, incremental updating, lightweight deployment strategies, and distributed detection schemes that preserve high detection capability, calibrated false alarm control, and explainability under real-time constraints.

In summary, this study establishes a solid foundation for explainable and deployment-aware IoT attack detection while also highlighting open challenges in cross-dataset generalization, threshold calibration, operational adaptation, and human-centered explainability. These directions provide a clear path for extending the present work toward more adaptive, transferable, and operationally grounded intrusion detection systems.

## Figures and Tables

**Table 1:** Comparison of representative related studies and the present work.

Study	Dataset/Domain	Main Model Type	Explainability	Evaluation Setting	Fair-Budget Comparison	Unseen/Zero-Day-like Evaluation	Main Difference from This Work
Pinto Neto et al. [1]	CICIoT2023/IoT	Benchmark ML and DL models	No dedicated XAI framework	Standard benchmark evaluation	No	No	Provides the dataset basis but not the proposed framework
Chen and Guestrin [5]	General tabular learning	Gradient tree boosting	No	General supervised learning benchmarks	No	No	Establishes XGBoost foundation, not IoT IDS
Lundberg and Lee [7]	General interpretable ML	Additive attribution	SHAP	General interpretability framework	No	No	Provides theoretical basis for attribution
Arreche et al. [16]	IDS/cybersecurity	Explainable IDS framework	Yes	Standard IDS evaluation	No	No	Focuses on explainable IDS, not unseen-family IoT evaluation
Alabbadi and Bajaber [18]	IoT data streams	XAI-based IDS	Yes	Stream-based IoT IDS evaluation	No	No	Explores explainable IoT IDS, not fair-budget baseline comparison
Hu et al. [6]	IoT networks	XGBoost	Yes	Conventional IoT IDS evaluation	No	No	Closest XGBoost-related study, but no unseen-family protocol
Ha and Kim [23]	Zero-day IDS	ML-based IDS	Limited	Operationally constrained zero-day evaluation	No	Yes	Emphasizes zero-day realism, but not IoT XGBoost explainability
This work	CICIoT2023/IoT	Optimized XGBoost with multi-model baselines	XGBoost-native SHAP-compatible contribution analysis	Closed-world CV + unseen-family evaluation	Yes	Yes	Integrates fair comparison, unseen-family assessment, and XAI in one framework

**Table 2:** Dataset scale distribution used in this study.

Item	Value
Total samples	46,686,579
Total features	46
Benign samples	1,098,195
Attack samples	45,588,384
Attack ratio	97.65%
Number of malicious families for unseen-family evaluation	9

**Table 3:** Attack family distribution used in this study.

Attack Family	Number of Samples
DDoS	33,984,560
DoS	8,090,738
Mirai	2,634,124
Spoofing	486,504
Recon	354,565
Web	15,752
BruteForce	13,064
BrowserHijacking	5859
Backdoor	3218
Benign	1,098,195

**Table 4:** Models included in the experimental comparison and their roles.

Model	Category	Role in This Study
Default XGBoost	Gradient boosting tree	Reference boosting baseline
Optimized XGBoost	Gradient boosting tree	Proposed primary model
Random Forest	Bagging tree ensemble	Strong classical ensemble baseline
LightGBM	Gradient boosting tree	Efficient boosting baseline
CatBoost	Gradient boosting tree	Robust boosting baseline
Logistic Regression	Linear classifier	Linear baseline
Simple MLP	Neural network	Lightweight deep learning baseline

**Table 5:** Training budget design used in the experiments.

Experimental Suite	Models Included	Training Budget
Fair-budget baseline suite	All seven models	2,000,000 samples per model
Supplementary full-scale suite	Optimized XGBoost only	Full-scale training
Repeated unseen-family calibration suite	Six main tabular baselines	2,000,000 samples per run, repeated across five seeds
Threshold-calibrated suite	Six main tabular baselines	Validation-calibrated operating thresholds under fixed-FAR targets

**Table 6:** Fair-budget closed-world performance comparison across all models.

Model	Macro-F1	Balanced Accuracy	MCC	FAR	FNR	ROC-AUC	PR-AUC
Random Forest	0.9713	0.9743	0.9426	0.0499	0.0015	0.999702	0.999993
LightGBM	0.9602	0.9956	0.9230	0.0050	0.0039	0.999638	0.999991
Optimized XGBoost	0.9566	0.9945	0.9161	0.0067	0.0043	0.999556	0.999989
Default XGBoost	0.9449	0.9962	0.8952	0.0018	0.0057	0.999505	0.999988
CatBoost	0.9444	0.9962	0.8942	0.0019	0.0058	0.999480	0.999988
Simple MLP	0.9047	0.9942	0.8247	0.0006	0.0109	0.998592	0.999966
Logistic Regression	0.7431	0.9760	0.5708	0.0019	0.0462	0.992962	0.999820

**Table 7:** Fold-level stability of fair-budget closed-world performance.

Model	Macro-F1 (Mean ± Std)	Balanced Accuracy (Mean ± Std)	MCC (Mean ± Std)
Random Forest	0.9713 ± 0.0002	0.9743 ± 0.0004	0.9426 ± 0.0003
LightGBM	0.9602 ± 0.0020	0.9956 ± 0.0002	0.9230 ± 0.0038
Optimized XGBoost	0.9566 ± 0.0006	0.9945 ± 0.0002	0.9161 ± 0.0011
Default XGBoost	0.9449 ± 0.0006	0.9962 ± 0.0001	0.8952 ± 0.0011
CatBoost	0.9444 ± 0.0017	0.9962 ± 0.0001	0.8942 ± 0.0030
Simple MLP	0.9047 ± 0.0011	0.9942 ± 0.0001	0.8247 ± 0.0019
Logistic Regression	0.7431 ± 0.0012	0.9760 ± 0.0001	0.5708 ± 0.0017

**Table 8:** Fair-budget unseen attack family performance comparison.

Model	Macro-F1 Mean	Balanced Accuracy Mean	MCC Mean	FAR Mean	FNR Mean	ROC-AUC Mean	PR-AUC Mean
Random Forest	0.8433	0.9376	0.7295	0.0536	0.0712	0.986223	0.927317
Optimized XGBoost	0.8194	0.8459	0.7067	0.0086	0.2996	0.959919	0.885837
LightGBM	0.8158	0.8360	0.6902	0.0071	0.3209	0.936476	0.844976
CatBoost	0.7989	0.8060	0.6911	0.0018	0.3863	0.946336	0.877325
Default XGBoost	0.7973	0.8040	0.6884	0.0019	0.3901	0.957738	0.880816
Simple MLP	0.6689	0.7006	0.4857	0.0008	0.5979	0.911237	0.712945
Logistic Regression	0.5804	0.6754	0.3033	0.0024	0.6468	0.820052	0.564797

**Table 9:** Family-wise macro-F1 of selected models under the fair-budget unseen attack family evaluation.

Held-Out Family	Default XGBoost	Optimized XGBoost	Random Forest	LightGBM	CatBoost
Backdoor	0.8666	0.8175	0.6429	0.8312	0.8733
BrowserHijacking	0.8496	0.8357	0.7080	0.8345	0.8439
BruteForce	0.6736	0.7813	0.7948	0.6109	0.6878
DDoS	0.9984	0.9969	0.9862	0.9987	0.9992
DoS	0.9994	0.9975	0.9840	0.9983	0.9995
Mirai	0.9994	0.9975	0.9832	0.9976	0.9993
Recon	0.6277	0.6941	0.8670	0.7299	0.6506
Spoofing	0.3496	0.3960	0.7914	0.4792	0.3306
Web	0.8117	0.8579	0.8326	0.8619	0.8059

**Table 10:** Comparison between fair-budget and full-scale optimized XGBoost.

Setting	Protocol	Macro-F1	Balanced Accuracy	MCC	FAR	FNR	ROC-AUC	PR-AUC
Fair-budget optimized XGBoost	Closed-world	0.9566	0.9945	0.9161	0.0067	0.0043	0.999556	0.999989
Full-scale optimized XGBoost	Closed-world	0.9560	0.9969	0.9155	0.0017	0.0045	0.999689	0.999993
Fair-budget optimized XGBoost	Unseen-family mean	0.8194	0.8459	0.7067	0.0086	0.2996	0.959919	0.885837
Full-scale optimized XGBoost	Unseen-family mean	0.8300	0.8323	0.7344	0.0021	0.3334	0.965272	0.917757

**Table 11:** Requirements for moving from model-centered attribution to analyst-centered explanation.

Requirement	Operational Purpose	Example Evaluation Criterion
Alert narrative	Translate feature contributions into analyst-readable evidence.	Analyst-rated clarity and usefulness.
Traffic evidence view	Connect explanation outputs to raw or aggregated traffic behavior.	Ability to verify why an alert was triggered.
Historical comparison	Show whether the current event resembles prior benign or malicious cases.	Improved false-positive review efficiency.
Case-specific explanation	Provide separate interpretation workflows for TP, FP, FN, and TN cases.	Better error diagnosis and blind-spot identification.
Threshold context	Show how each alert relates to the selected FAR/FNR operating objective.	Improved understanding of deployment trade-offs.
Human-centered evaluation	Test whether explanations help actual analysts in realistic triage tasks.	Triage time, trust calibration, decision consistency, and investigation accuracy.

**Table 12:** Main strengths and limitations of the proposed study.

Aspect	Strength	Remaining Limitation
Dataset scale	Very large IoT cybersecurity dataset with diverse malicious traffic	Still limited to a single benchmark
Evaluation design	Includes closed-world, unseen-family, repeated-sampling, and fixed-FAR evaluation	Unseen-family evaluation is not identical to full open-world zero-day evaluation
Baseline comparison	Fair-budget comparison across seven models	Full-scale comparison was only applied to optimized XGBoost
Sampling reproducibility	Uses stratified sampling without replacement and split-overlap audit	Additional cross-dataset sampling validation is still needed
Detection task	Clear binary malicious-versus-benign formulation	Does not cover multiclass attack attribution
Threshold analysis	Includes validation-based fixed-FAR and cost-sensitive threshold analysis	Real deployment still requires drift-aware and online calibration
Explainability	Includes global and local XAI-based contribution analysis	Uses XGBoost native pred_contribs rather than a fully harmonized comparison across explanation backends
Practical relevance	Considers false alarms, false negatives, threshold calibration, and interpretability	User-centered explanation evaluation remains future work

## Data Availability

The CICIoT2023 dataset used in this study is publicly available from the Canadian Institute for Cybersecurity. The processed experimental outputs and supplementary analysis files are available from the author upon reasonable request. The preprocessing scripts, model training configuration, and evaluation scripts are available from the corresponding author upon reasonable request.

## Supplementary Materials

The following supporting information can be downloaded at: https://www.mdpi.com/article/10.3390/s26103005/s1, Table S1: Sampling and split audit summary for repeated unseen-family evaluation; Table S2: Optimized XGBoost hyperparameter configuration; Table S3: Notation and metric glossary; Table S4: Repeated default-threshold unseen-family results over 45 runs; Table S5: Validation-based threshold calibration at approximate FAR target = 0.01; Table S6: Top 10 global feature contributions of optimized XGBoost; Table S7: Local reconstruction check for XGBoost native pred_contribs.
