# Supplementary material for: An Explainable XGBoost-Based Framework for IoT Attack Detection with Unseen Attack Family Evaluation
Source: Sensors (Basel). 2026 May 10;26(10):3005. doi: 10.3390/s26103005 (PMC13210460; doi:10.3390/s26103005)
Supplement: Supplementary file 1 [file sensors-26-03005-s001.zip › sensors-4270515-supplementary.pdf]

Supplementary Materials

Table S1. Sampling and split audit summary for repeated unseen-family evaluation.

Item	Value
Repeated seeds	5
Held-out malicious families	9
Total repeated unseen-family runs	45
Training budget per run	2,000,000 samples
Fitting subset per run	1,700,000 samples
Validation subset per run	300,000 samples
Benign test samples per run	219,639
Mean unseen-family attack test samples	5,065,376
Sampling method	Stratified sampling without replacement from the train candidate pool
Train–test overlap	0 in all 45 runs
Fit-validation overlap	0 in all 45 runs
Fit-test overlap	0 in all 45 runs
Validation-test overlap	0 in all 45 runs

Table S2. Optimized XGBoost hyperparameter configuration.

Parameter	Value
n_estimators	354
max_depth	10
learning_rate	0.170176
subsample	0.799013
colsample_bytree	0.808141
min_child_weight	1
gamma	0
reg_lambda	1
reg_alpha	0
default threshold	0.5
fixed-FAR validation target	approximately 0.01
validation split	300,000 samples from the fair-budget training subset
random seed handling	Repeated unseen-family evaluation over five seeds

Table S3. Notation and metric glossary.

Symbol/Term	Full Name	Definition	Interpretation
TP	True Positive	Attack traffic correctly classified as attack	Correct attack detection
TN	True Negative	Benign traffic correctly classified as benign	Correct benign classification
FP	False Positive	Benign traffic incorrectly classified as attack	False alarm
FN	False Negative	Attack traffic incorrectly classified as benign	Missed attack
FAR	False Alarm Rate	FP/(FP + TN)	Proportion of genuine benign samples misclassified as attacks
FNR	False Negative Rate	FN/(FN + TP)	Proportion of attack samples missed by the detector
MCC	Matthews Correlation Coefficient	Correlation coefficient over TP, TN, FP, and FN	Robust binary classification measure under imbalance

Macro-F1	Macro-averaged F1-score	Mean of benign-class F1 and attack-class F1	Gives equal weight to benign and attack classes
Balanced Accuracy	Balanced Accuracy	(TPR + TNR)/2	Average of attack recall and benign specificity
PR-AUC	Precision-Recall AUC	Area under the precision-recall curve	Ranking quality with attack as positive class
IAT	Inter-Arrival Time	Time interval-related traffic feature	Captures temporal behavior
XAI	Explainable Artificial Intelligence	Explanation-oriented model analysis	Supports model inspection and diagnostic interpretation

Table S4. Repeated default-threshold unseen-family results over 45 runs.

Model	Macro-F1 Mean ± Std	MCC Mean ± Std	FAR Mean ± Std	FNR Mean ± Std	Attack Recall Mean
Random Forest	0.8415 ± 0.1182	0.7265 ± 0.1945	0.0551 ± 0.0116	0.0712 ± 0.0930	0.9288
Optimized XGBoost	0.8196 ± 0.1812	0.7075 ± 0.2425	0.0090 ± 0.0026	0.2976 ± 0.2697	0.7024
LightGBM	0.8174 ± 0.1690	0.6926 ± 0.2550	0.0077 ± 0.0017	0.3151 ± 0.2862	0.6849
Default XGBoost	0.7999 ± 0.2026	0.6906 ± 0.2683	0.0022 ± 0.0006	0.3842 ± 0.3152	0.6158
CatBoost	0.7981 ± 0.2043	0.6879 ± 0.2705	0.0023 ± 0.0007	0.3839 ± 0.3169	0.6161
Logistic Regression	0.5845 ± 0.2040	0.3134 ± 0.3125	0.0024 ± 0.0007	0.6422 ± 0.4425	0.3578

Table S5. Validation-based threshold calibration at approximate FAR target = 0.01.

Model	Observed FAR	Macro-F1	MCC	FNR	Attack Recall	Mean Threshold
Random Forest	0.0102	0.8754	0.7757	0.2000	0.8000	0.7759
Optimized XGBoost	0.0105	0.8323	0.7193	0.2760	0.7240	0.4327
Default XGBoost	0.0106	0.8318	0.7163	0.2800	0.7200	0.1668
CatBoost	0.0104	0.8299	0.7121	0.2856	0.7144	0.1488
LightGBM	0.0104	0.8240	0.6976	0.2981	0.7019	0.3920
Logistic Regression	0.0100	0.6100	0.3368	0.6119	0.3881	0.3639

Table S6. Top 10 global feature contributions of optimized XGBoost.

Rank	Feature	Mean Absolute Contribution
1	rst_count	3.0979
2	IAT	3.0435
3	urg_count	1.1092
4	Tot size	1.0911
5	Number	0.7773
6	Header_Length	0.7388
7	Magnitude	0.7295
8	Protocol Type	0.4535
9	flow_duration	0.4350
10	Rate	0.4120

Table S7. Local reconstruction check for XGBoost native pred_contribs.

Case	Model Probability	Probability from Reconstructed Margin	Absolute Probability Gap
TP	1.000000	0.99999996	4.35×10^{-8}
FP	0.500298	0.50029809	1.71×10^{-7}
FN	0.499980	0.49998004	6.71×10^{-8}
TN	0.00000085	0.00000085	4.96×10^{-12}
